# Computational Analysis of Dynamic Light Exposure of Unicellular Algal Cells in a Flat-Panel Photobioreactor to Support Light-Induced CO_2_ Bioprocess Development

**DOI:** 10.3389/fmicb.2021.639482

**Published:** 2021-04-01

**Authors:** Nicolò S. Vasile, Alessandro Cordara, Giulia Usai, Angela Re

**Affiliations:** ^1^Centre for Sustainable Future Technologies, Fondazione Istituto Italiano di Tecnologia, Genova, Italy; ^2^Department of Applied Science and Technology, Politecnico di Torino, Torino, Italy

**Keywords:** computational fluid dynamics, particle tracing, carbon dioxide bioconversion, algal bioprocess, simulation modeling, photobioreactor, light distribution analysis, *Synechocystis* sp. PCC 6803

## Abstract

Cyanobacterial cell factories trace a vibrant pathway to climate change neutrality and sustainable development owing to their ability to turn carbon dioxide-rich waste into a broad portfolio of renewable compounds, which are deemed valuable in green chemistry cross-sectorial applications. Cell factory design requires to define the optimal operational and cultivation conditions. The paramount parameter in biomass cultivation in photobioreactors is the light intensity since it impacts cellular physiology and productivity. Our modeling framework provides a basis for the predictive control of light-limited, light-saturated, and light-inhibited growth of the *Synechocystis* sp. PCC 6803 model organism in a flat-panel photobioreactor. The model here presented couples computational fluid dynamics, light transmission, kinetic modeling, and the reconstruction of single cell trajectories in differently irradiated areas of the photobioreactor to relate key physiological parameters to the multi-faceted processes occurring in the cultivation environment. Furthermore, our analysis highlights the need for properly constraining the model with decisive qualitative and quantitative data related to light calibration and light measurements both at the inlet and outlet of the photobioreactor in order to boost the accuracy and extrapolation capabilities of the model.

## Introduction

One of the most compelling long-term global sustainability goals is not just to abate the emissions of greenhouse gasses, but also to substitute environmentally expensive processes based on fossil fuels with biobased sustainable alternatives (Sustainabledevelopment Organization, 2015). Carbon dioxide (CO_2_) sequestration and transformation using microorganisms as catalysts could lead to breakthroughs in CO_2_ capture and utilization (Lorenzo et al., 2018; McCarty and Ledesma-Amaro, 2019). Photosynthetic microorganisms have garnered an enormous interest since they can be repurposed to convert atmospheric carbon dioxide and renewable electricity-based light, acting, respectively, as carbon and energy sources, into biobased value-added compounds (Luan and Lu, 2018; van den Berg et al., 2019). Indeed, the biosynthesized compounds can be functionalized in the transport (Lan and Liao, 2012; Gao et al., 2017b; Vidal, 2017; Shabestary et al., 2018; Liu et al., 2019; Wang L. et al., 2020) and energy (Saper et al., 2018) sectors as well as in the packaging (Ni et al., 2016; Nozzi et al., 2017; Yao et al., 2020), health (Lin et al., 2019; Dienst et al., 2020), cosmetic and personal care sectors (Choi et al., 2016; Derikvand et al., 2017), and in the food industry (Caporgno and Mathys, 2018; Bernaerts et al., 2019; Grossmann et al., 2020) among others. The synthetic biology toolkit to unlock the potential of cyanobacterial cell factories has substantially increased in recent years (Taton et al., 2014; Wendt et al., 2016; Vogel et al., 2017; Janasch et al., 2018; Santos-Merino et al., 2019; Vasudevan et al., 2019; Vavitsas et al., 2019). Nonetheless, a great deal of improvement is still needed to simplify and accelerate the transfer of bench-scale bioproduction processes into commercial plants (Jones, 2014; Johnson et al., 2018; Gifuni et al., 2019; Xia et al., 2019). In addition to the availability of metabolic engineering tools, the advantageous factors are cyanobacterial genetic malleability (Xiong et al., 2017), competitive carbon conversion efficiency (Pérez et al., 2019), and native ability to grow in a very poor culture medium (Jahn et al., 2018). These biocatalysts could integrate into climate-mitigating industrial pipelines and sustainably fuel the circular bioeconomy. The potential of photoautotrophic microorganisms such as cyanobacteria to pursue the biobased production of marketable products is being exploited by several companies developing renewable fuels (Farrokh et al., 2019; Yen et al., 2019) and developing innovative solutions to supply bio-based chemical intermediates in green chemistry formulations (Corbion, 2020; Cyano Biotech GmbH, 2020; Photanol, 2020; Pond Tech, 2020).

Efforts are being intensified to enhance the operating reliability of the newly developed biotechnologies (Gifuni et al., 2019). In this perspective, also, a large number of computational approaches are now used to achieve an enhanced understanding (Asplund-Samuelsson et al., 2018; Broddrick et al., 2019), analytical quantification (Zavřel et al., 2019), and control of bioprocesses (Narayanan et al., 2020). In addition to approaches accounting for the multi-factorial design of experiments (Kommareddy and Anderson, 2013), there exists a plethora of computational simulation approaches in process design such as multi-scale physical models (Pruvost and Cornet, 2012; Solimeno et al., 2017; Weise et al., 2019), coupling computational fluid dynamics and kinetic modeling (Perner-Nochta and Posten, 2007; Seo et al., 2014; Loomba et al., 2018; Scheufele et al., 2019), and artificial intelligence-based models (Rio-Chanona et al., 2016, Rio-Chanona et al., 2019).

In the drive to identify the conditions maximizing the productivity of a photobioreactor as a whole, it is necessary to understand the coupling among hydrodynamics and mass transport, radiation, and cellular growth kinetics. Modeling approaches, which invoke excessively restrictive assumptions, such as mono-dimensionality in light transmission (Beer-Lamber law and its variants) or perfect mixing (Carvalho and Malcata, 2003; Krujatz et al., 2015), oversimplify the problem and are bound to provide scarce predictive accuracy (Csgör et al., 2001; Straub, 2011). The variation over time of the environmental physical features to which cells are exposed has to be accounted for. Indeed, flow hydrodynamics influences the availability of substrates for the cells and the history of cells exposure to light. Therefore, several approaches, such as the Lagrangian and Eulerian simulation approaches, have been developed to couple fluid dynamics and radiation transport with cell growth (Gao et al., 2018). In some articles, simulations are performed with simplifying assumptions with respect to the geometry, thus, simulating the phenomena internal to the reactor in 1D (Koller et al., 2017) or 2D (Wheaton and Krishnamoorthy, 2012) configurations, while in the most complete articles, the models are developed in a 3D configuration (Solimeno et al., 2015; Loomba et al., 2018). As for the analysis of the phenomena principally analyzed in our article, i.e., those related to light transmission, many different simulation scenarios are reported in literature. In some cases, the transmission of light radiation is supposed to be monodirectional (Zhang et al., 2015; Naderi et al., 2017), while in other cases, like ours, all possible directions of propagation are considered (Zhang et al., 2015). Furthermore, it is worthwhile to note that the culture medium is multiphasic as it includes a liquid (nutrients, dissolved reagents, and reaction products), gaseous (bubbles), and solid phase. Hence, in a faithful reproduction of light transmission, it is appropriate to consider the specific effects of the different components with the help of different parameters related to the absorption and scattering of the light. However, the medium is usually assumed to exhibit a single liquid phase. As a consequence of this unrealistic assumption, the effect of light scattering/absorption due to bubbles (Csgör et al., 2001; Zhang et al., 2015) is not accounted for. Similarly neglected is the effect of light absorption by particles (Seo et al., 2014; Solimeno et al., 2017), which induces the classical shadow effect, decreasing the amount of light available to the bacterial cells as one moves away from the light source. Our modeling approach, instead, explicitly accounts for the multiphysical nature of the environment within the reactor. When these phenomena are considered, the related coefficients are usually set according to the literature (Fernández et al., 2012; Naderi et al., 2017), whereas in our study, they are fitted from experimental data. The correct modeling reproduction of the light pattern makes it possible to improve the accuracy in simulating the biochemical phenomena taking place inside the reactor (Fernández et al., 2012), and to correlate bacterial growth and its possible limitation and inhibition with light transmission (Huang et al., 2012; Koller et al., 2017; Zhang et al., 2017).

This study proposes a 3D multi-physics mathematical model of a flat-panel photobioreactor, developed on the Comsol^®^ platform, which enables the comprehensive simulation of different phenomena such as thermo-fluid dynamics, cyanobacterial growth kinetics, mass transfer, and light transmission by formulating the corresponding equations. The discrete ordinates method was used to calculate the light radiation in all the photobioreactor domains for each time step. Computing particle trajectories was instrumental to characterize the exposure of individual cells to light, which primarily influences the growth rate, biomass concentration, and ultimately, biomass productivity. Local light radiation profiles were simulated in order to assess the amount of light that is actually perceived and absorbed by bacterial cells in relation to different illumination conditions. The validity of the model was examined by comparing model predictions with direct measurements of key process parameters. Here, with our approach, we could appraise the remarkable difference between the externally supplied light and the light received by the cells and we could deepen the plurality of the underlying reasons. We linked the study of light transmission inside the PBR vessel and the exploration of the residence time of bacterial cells in different PBR domains to the experimental observation of cyanobacterial physiological parameters. Our modeling framework is able to couple the reconstruction of single cell trajectories across differently irradiated PBR zones with cellular growth kinetics. Therefore, our simulation framework is exploitable to screen and identify the operating conditions of the photobioreactor which optimize the accumulation of productive algal biomass.

The understanding generated by our model, which unveils otherwise inaccessible characteristics of an artificially lit photoautotrophic cultivation environment, allows guiding the PBR optimization toward an enhanced biomass photosynthetic growth efficiency and productivity. Nonetheless, the complete realization of the extrapolation capabilities of a modeling approach requires the model to be a representative of the system of interest. In this regard, we argue that, as a prerequisite of the implementation of effective modeling, it is crucial for the model to be backed by consistent process data acquisition and management. We show, with a tailored experimental design, how the predictions of relevant process characteristics resulting from the model depend on the existence of accurate information concerning both the light entering and the light leaving the photobioreactor.

To unveil the interrelationships between model predictions and the nature of light at the entrance of the photobioreactor, our experimental plan varied the intensity of the light supplied to the photobioreactor from 50 to 1,200 μE every 24 h under three different types of calibration of the LED light source. According to our results, the initialization of the source of light at the entrance of the photobioreactor should not be limited to the prearranged light intensity but should also account for the effects related to the specific calibration procedure. Indeed, our results demonstrated that setting the model with the light resulting from different calibrating configurations impacts differently on the local light distribution inside the photobioreactor and thus on the light that is actually perceived by the bacteria, obviously influencing microorganism growth kinetics.

Alongside a careful definition of the light entering the photobioreactor, we demonstrated the benefits of accompanying the modeling with measurements of light intensity at the exit of the photobioreactor under both abiotic and biotic culture conditions. Indeed, informing the model with experimental data acquired in the absence/presence of photosynthetic cells facilitates the estimation of the interconnections between local environmental traits and *Synechocystis* physiological and growth attributes.

This work proposes an innovative modeling framework which allows gaining insights in the complex relationships between several light characteristics and cell physiology. Furthermore, our analysis highlights the opportunities and challenges in properly constraining the model with decisive qualitative and quantitative data related to the light at the entrance and at the exit of the photobioreactor.

## Materials and Methods

### Pre-cultures Conditions

For all the experiments, the pre-cultures of glucose-tolerant wild-type *Synechocystis* sp. PCC6803, kindly provided by Devaki Bhaya (Department of Plant Biology, Carnegie Institution for Science, Stanford, CA, United States), were prepared by growing cells in flasks in 25 ml of BG 11 medium with a modified recipe as described in van Alphen and Hellingwerf (2015).

Pre-cultures were grown for 1 week at 30°C in a shaking incubator at 120 rmp (Innova 44, New Brunswick Scientific, Edison, United States) under constant white light illumination at approximately 50 μmolphotons m^–2^s^–1^ (μE), measured with the photometer (2102.2, Delta Ohm s.r.l, Padua, Italy).

### PBR Growth Conditions

*Synechocystis* pre-culture was used to seed a temperature-controlled flat panel photobioreactor (PBR)—model FMT150.2/400 (Photon Systems Instruments, Drasov, Czech Republic) (Nedbal et al., 2008)—with 20 ml of culture. This study was performed in a customized 380 ml vessel for FMT150 (Supplementary Figure 1). Cells were grown in the BG11 medium modified as described above and supplemented by 10 mM of NaCHO3.

The lid of the PBR held a combined pH/temperature probe and a Clark-type dissolved O_2_ (dO_2_) probe (Photon Systems Instruments, Drasov, Czech Republic). The optical density (OD) was measured by an integrated densitometer at 720 and 680 nm. The temperature was settled at 30 ± 1°C and pH ∼ 8.

A constant supply of CO_2_ was provided by sparging the medium with 1% CO_2_ (v/v) in N_2_. A gas mixer (GMS150 micro, Photon Systems Instruments, Drásov, Czech Republic) coupled to a mass flow controller (EL-FLOW prestige FG-201CV, Bronkhorst High-Tech BV, AK Ruurlo, Netherland) were employed to supply 150 ml/min of gas mixture to the PBR.

**FIGURE 1:**
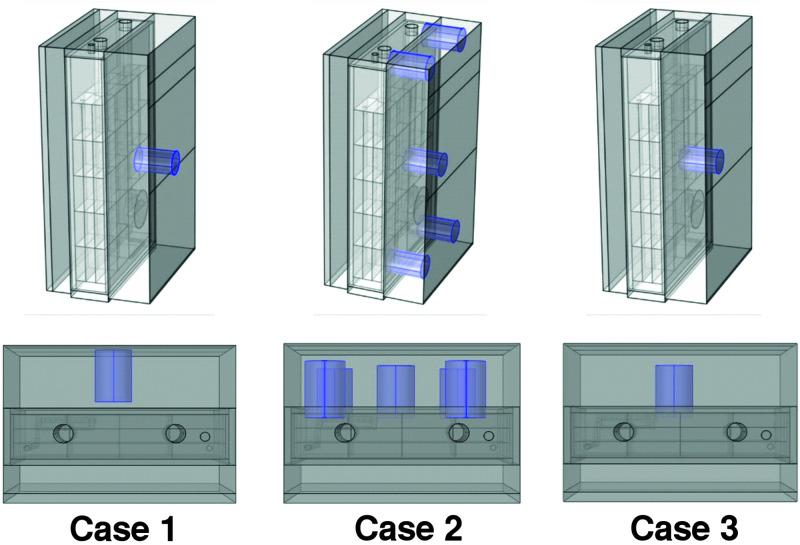
Positioning of the PAR probe for the three calibration procedures. Calibration cases 1, 2, and 3 are shown from left to right. In case 1, the light sensor was positioned on the LED light panel and a single measure was acquired in the middle position of the panel. In case 2, the light sensor was positioned at 1 cm from the LED panel and the measurements acquired in the central position and in four angular positions. In case 3, the light sensor was positioned at 1 cm from the LED panel and a single measure was acquired in the middle position.

The cellular suspension was illuminated from one side of the PBR by orange–red light (636 nm) emitting diodes (LEDs). The light calibration was performed with the assistance of the PBR software and the use of a light photometer (2102.2 photometer, Delta Ohm s.r.l, Padua, Italy), as shown in Supplementary Figure 2. For each VDC value (0–100%) set by the PBR software, we adopted three different types of calibration of the LED panel, which differed in the relative distances between the light meter and the LED panel and in the position(s) selected for acquiring the light intensity measurement(s). We refer to the three calibration setups under consideration in our study as case 1, case 2, and case 3 (Figure 1). More in detail, in case 1, the light sensor was positioned on the LED light panel and a single measure was acquired in the middle position of the panel. In case 2, the light sensor was positioned at 1 cm from the LED panel and the measurements acquired in the central position and in four angular positions (Figure 1) were averaged, Finally, in case 3, the light sensor was positioned at 1 cm from the LED panel and a single measure was acquired in the middle position.

For each configuration tested in the calibration stage, cells were grown at 50, 100, 200, 300, 500, 950, and 1,200 μmolphotons m^–2^s^–1^. Cyanobacterial cultures were subject to increasing light intensities every 24 h and each light regime was kept for at least 24 h as this time frame was found to be long enough for reaching a stable growth rate and dissolved oxygen in the culture medium (Supplementary Figure 3). For each light regime settled, the light in output from the PBR was measured at the central and angular positions, both in the presence and absence of the *Synechocystis* culture. Experiments were performed in three replicates for calibration case 1 and in two replicates for calibration cases 2 and 3.

The PBR was run in a semicontinuous mode by controlled dilution (turbidostat) of the growing cell suspension. Dilution was based on the changes at OD720, measured by the integrated densitometer and calibrated to the benchtop spectrometer OD730 to maintain the OD730 approximately at 0.4 ± 2.5%. Dilution was performed by a peristaltic pump automatically controlled by the software of the photobioreactor. The range of 2.5% was intentionally chosen to be large enough to allow the software to calculate the cells growth rate from the curve of the OD slope between the dilutions.

### Dry Weight

For the determination of the dry cell weight, at the end of each 24 h step light increment, an aliquot of 20 ml of culture was harvested in a sterile 50 ml falcon tube. The suspension was pelleted at 1,500× g at room temperature for 15 min. The supernatant was gently removed, and the pellet was delicately washed with 2 ml of sterilized MilliQ water to remove medium salts. The sample was again pelleted at 1,500× g for 15 min, the supernatant was carefully removed and other 2 ml of MilliQ distilled water were used to resuspend the pellet in a preweighted 2 ml eppendorf. After centrifugation (1,500× g for 15 min), the supernatant was discarded. The tubes were subsequently dried overnight in a stove at 90°C and finally weighted again. In parallel, the OD730 of the sampled cells was measured with a Bench spectrophotometer (7315, Jenway, Staffordshire, England) and used to normalize the dry cell weight per OD730 (Andreas Angermayr et al., 2016; Du et al., 2016; Cordara et al., 2018).

### Determination of Photosynthetic Efficiency

The photosynthetic efficiency was calculated as grams of biomass formed per mole of photons (van Alphen and Hellingwerf, 2015; Luimstra et al., 2019). We calculated the amount of light available to the culture as the difference between the light in input to the PBR, which we called *I*_*s*,*in*_, and the light remaining after the passage through the PBR, which we called *I*_*out*_. *I*_out_ was both experimentally measured and calculated through model simulations in order to calculate the amount of light absorbed in the reactor volume. We used the growth rate and cell dry weight values to calculate the amount of biomass produced in the actual volume during 24 h for each *I*_*s*,in_ used.

### Mathematical Model Description

The 3D multi-physics, multi-component, multi-phase, and not isothermal model of the photobioreactor was developed on the COMSOL 5.5^®^ platform and allowed us to simulate different phenomena such as fluid dynamic, heat transfer and radiation in different media, bacterial growth kinetics, gas-liquid mass transfer, transport of species, and particle tracing by formulating the corresponding equations. Supplementary Table 1 reports the equations sets described in detail in Cordara et al. (2018). Particle tracing related equations are described separately in the following.

Supplementary Figure 4A shows the general design of the 3D model based on the PBR geometry. It is worth noting that our model accounts also for the presence of the glass and probes for O_2_ and pH inside the liquid mixture (Supplementary Figure 1D) and their effect on light transmission. Table 1 contains the geometrical parameters of the vessel.

**TABLE 1:** Geometrical parameters of the PBR and its components.

Domain	Element size	[m]
Reator	Height	0.1983
	Width	0.11
	Thickness	0.024
Sparger	Diameter of inlet	0.002
	Diameter of holes	0.0004
	Number of holes	7
	Lenght	0.03
Anchor	Diameter	0.006
	Lenght	0.035
Vessel	Glass Thickness	0.0033

Free tetrahedral meshing was applied to the created model prior to analysis (Supplementary Figure 4B). Meshing size (1,253,654) was selected in order to prevent model inaccuracy and imprecision resulting from model meshing. In fact, with this huge number of tetrahedral elements we are able to perform the calculations with an adequate degree of convergence of the results.

#### Particle Tracing

In addition to the previously considered equations, in this work, we also take into account the presence of solid particles. The assumptions underlying this type of modeling are described as follows:

(i) the particles have a spherical geometric shape;
(ii) particle initial mass and density has been derived from the experimental results;
(iii) particle motion inside the reactor is straightly linked to the fluid-dynamic calculations;
(iv) the reactions take place at the bacterium/liquid mixture interface;
(v) particle growth during the cultivation period is calculated through Eq. (33) in Supplementary Table 1 and is homogeneous for all the particles.

The particle trajectory and physical properties in the photobioreactor is determined by the particle tracing module for the fluid flow interface in Comsol^®^. In this module, the motion, *v*_*p*_, of a particle with mass *m*_*p*_ in the vessel is described by Eq. (34) in Supplementary Table 1 (Seo et al., 2014).

(1) $$\frac{d}{d\,t}\,\left(m_{p}\,v_{p}\right)=F_{D}+F_{g}+F_{e\,x\,t}$$

where *F*_*D*_ and *F*_*g*_are, respectively, the drag and gravity force the particles are subjected to. *F*_*ext*_ stands for some additional (e.g., electric, magnetic) force acting on a particle. The particle momentum is defined by Newton’s second law, which states that the net force on a particle is equal to the time rate of change of its linear momentum in an inertial reference frame (Loomba et al., 2018).

The drag force is defined by Eq. (35) in Supplementary Table 1.

(2) $$F_{D}=m_{p}\,F_{d}\,\left(v-v_{p}\right)$$

where *F_*d*_ = 18η/(ρ_*p*_d_*p*_^2^)* is the drag force per unit mass, *ρ_*p*_* the particle density, and *d*_*p*_ the particle diameter. The gravity force is defined by Eq. (36) in Supplementary Table 1.

(3) $$F_{g}=m_{p}\,g\,\frac{\rho_{p}-\rho }{\rho_{p}}$$

From the particle velocity, its trajectory, *x*_*p*_, is determined by solving the differential equation

(4) $$\frac{d\,x_{p}}{d\,t}=v_{p}$$

When the particle mass is being computed, such that accretion or evaporation can take place, the mass is moved outside the time derivative to prevent non-physical acceleration of the particles. This assumption is that any mass lost by the particles continues to move with the particle velocity and does not cause the particle to decelerate.

We also consider particle-particle interactions to make particles exert forces on each other (linear elastic forces) as described by Eq. (38) in Supplementary Table 1.

(5) $$F=-k_{E\,L}\,\sum_{j=1}^{N}\left(\left|r-r_{j}\right|-r_{0}\right)\,\frac{r-r_{j}}{\left|r-r_{j}\right|}$$

Where *k*_*EL*_ is the spring constant (N/m), *r*_0_ (m) is the equilibrium distance between particles, and *r* (m) is the relative distance between particles.

## Results

Cyanobacteria are remarkably promising oxygenic phototrophic cell factories for manifold applications through the integration within innovative business models (Venkata Mohan et al., 2016).

Since the livelihood of cyanobacteria is directly dependent upon light, the technological exploitation of photosynthetic chassis strains is obligatorily reliant on a comprehensive investigation and understanding of the interrelationships between the irradiated light and the physiological traits of the cyanobacterial cells in the artificially lit cultivation environment (Andersson et al., 2019; Luimstra et al., 2019; Ho et al., 2020). To this aim, we designed an experimental campaign to explore the effects of illumination characteristics, including variation of orange-red light intensities and calibration procedures, on cyanobacterial physiology in a flat-panel photobioreactor. The experimental dataset on cyanobacterial physiology at varied illumination conditions was complemented with model simulations comprehensive of heat transfer with light transmission, fluid dynamics, and cellular growth kinetics. It was of utmost importance to acquire experimental data both in the absence and in the presence of cyanobacterial cells in order to facilitate the estimation of the radiation parameters employed in our modeling framework.

The exploration of the calibration effects on our simulation-aided analysis of the artificially lit flat-panel bioreactor envisaged three possible configurations thereafter referred to as calibration case 1, case 2, and case 3 and illustrated in Figure 1. Different from case 1, where the light sensor is placed on the LED panel, cases 2–3 envisage the light sensor at 1 cm from the LED panel. Cases 2 and 3 differ from each other by the number of measurement points used for light calibration, one central position and four angular positions for case 2 and one central position for case 3. To unveil the interrelationships between the illumination of the photobioreactor and the exposure of cyanobacterial cells to light, our experimental plan varied, for each type of calibration of the LED panel, the intensity of the light preset for the photobioreactor from 50 to 1,200 μE. The increase in the incident light intensity occurred every 24 h and the cell cultures were maintained at each light regime for at least 24 h. The duration of the exposition time at a certain lightening was indeed sufficient to achieve stable measurements of growth rate and dissolved oxygen in the culture medium (Supplementary Figure 3). For each light regime settled, the light in the output from the PBR was measured at the central and angular positions, both in the presence and in the absence of the *Synechocystis* culture.

### Model Accurately Predicts the Phototrophic Properties of the Cell Culture

**FIGURE 2:**
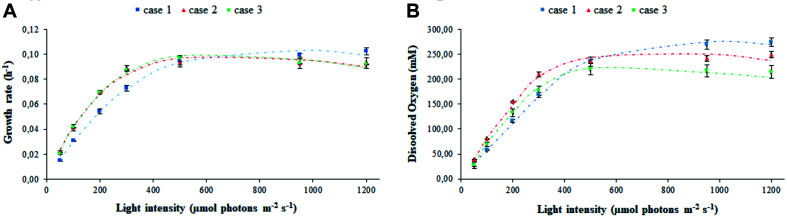
Calibration affects the assessment of photo-limited, photo-saturated, and photo-inhibited growth of cyanobacterial cells. Shown are the experimental (symbols) and simulated (dashed line) values of **(A)** growth rate and **(B)** oxygen released in the medium by *Synechocystis* at increasing light intensity *I_*s*,in_*, in relation to each calibration case. Symbols show the mean values over the biological replicates for each calibration case and are accompanied by their respective standard deviation bars.

The experimental values of the transmitted light and of the light detected at the vessel output, in biotic and abiotic conditions, have been used to fit the parameters related to light transmission in the various PBR domains taken into consideration, namely: air, vessel glass, stainless steel (wall, probes, and sparger), water with medium, bacteria, and gas bubbles (Supplementary Figure 1). The estimated parameters set was used to obtain the modeled trends for cyanobacterial growth rate (Figure 2A) and oxygen released in the medium (Figure 2B). The agreement between the experimental and simulated values with regard to both of these physiological traits demonstrates the solidity of the model built. The effect of different calibration approaches on growth rate μ and dissolved oxygen is neatly evident at the extreme light intensities which were set in our experiments. Specifically, Figure 2 shows the existence of opposite trends in the extreme regions corresponding to low incident light (I_*s*,in_ < 400 μE) and high incident light (I_*s*,in_ > 600 μE). In the intermediate region (400 < I_*s*,in_<600 μE), the values of μ and dissolved oxygen are almost uniform. In the low incident light region, we noticed a higher growth rate in the calibration cases 2–3 than in the case 1, with a more pronounced oxygen production for case 2 with respect to case 3. In the high incident light region, the trend is reversed with growth being faster in case 1 than in cases 2–3. This observation is due to the fact that in the calibration case 1, where the sensor is placed on the panel, the value of light intensity preset via software can be achieved by supplying less electrical power to the panel compared to cases 2–3, and that, as a consequence of it, LEDs emit at lower light intensity. When we place the sensor at 1 cm from the panel, according to the calibration cases 2–3, calibration is necessarily influenced by the absorption and scattering of light, which occurs in the space separating the sensor from the panel, and by the absorption of light by the surrounding black surfaces (Huang et al., 2011). Therefore, in the calibration cases 2–3, the LED panel has to be supplied with more electrical power and the LEDs have to emit light at a higher intensity to measure the prescribed light intensity in the sensor. Consequently, the light at which bacteria are exposed in the calibration case 1 is attenuated, when compared to the calibration cases 2–3. Since cyanobacterial photoinhibition is known to be induced by extreme high light intensity (Ogawa et al., 2018), it follows that, when the prescribed light intensity is low, bacteria grow faster (Figure 2A) and release more oxygen in the medium of the PBR (Figure 2B) in the calibration cases 2–3 than in the case 1. When the prescribed intensity is high, the physiological response of the bacterial culture is opposite: bacteria grow faster, and the dissolved oxygen registered in the medium is higher in the case 1 than in cases 2–3. Therefore, our experiments quantitatively show that calibration affects the physiological traits of the cyanobacterial culture in a non-negligeable manner.

**FIGURE 3:**
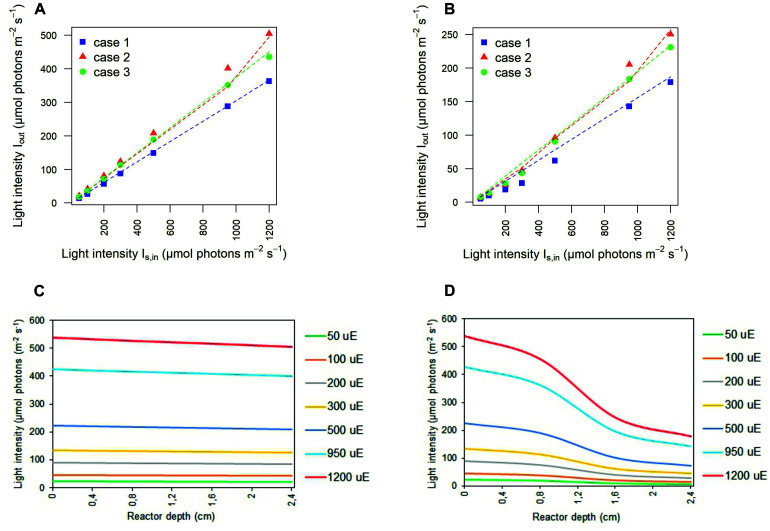
Simulated light intensities recapitulate experimental light intensities at the outlet of the photobioreactor. Comparison between experimental (symbols) and simulated (dashed line) light intensities at the outlet of the photobioreactor in correspondence to each calibration case at increasing light intensity *I_*s*,in_* in abiotic **(A)** and biotic **(B)** conditions. Symbols show the mean values over the biological replicates for each calibration case. The light transmitted along the PBR depth is attenuated as a result of cyanobacterial cells. 1D trend of simulated light intensity along the reactor depth for the calibration case 1, in abiotic **(C)** and biotic **(D)** configurations.

The values inferred for the light at the outlet of the photobioreactor were contrasted with those experimentally determined, in the abiotic (Figure 3A) and biotic (Figure 3B) cases in each calibration setup. In the abiotic condition, modeling effectiveness is largely insensitive to the calibration choice and the prescribed light intensity at the photobioreactor entrance. In the biotic condition, predictability was found to generally improve with increasing light intensity and to be influenced by the calibration choice. In this regard, our model is particularly effective in inferring the transmitted light in calibration case 2 whereby the light sensor is placed at 1 cm from the panel of LEDs and the light intensities measurements at the central and angular positions are averaged.

**FIGURE 4:**
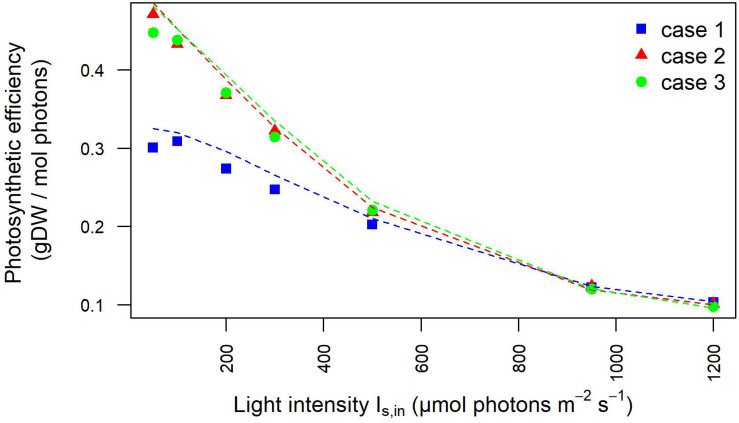
Photosynthetic efficiency dependency on light intensity is influenced by calibration. Photosynthetic efficiency is displayed at increasing light intensity under each calibration case. The values derived from experimentally determined light intensities at the inlet and outlet of the photobioreactor are shown by symbols. The photosynthetic efficiency values derived from simulated light intensities at the inlet/outlet of the photobioreactor are shown by dotted lines.

Moreover, the outlet light intensities derived from our model simulations were employed to estimate the light-dependent photosynthetic efficiency of the photobioreactor in terms of grams of biomass produced per mole of photons available in the photobioreactor. The full agreement with photosynthetic efficiency values derived from experimentally acquired outlet light intensities confirmed the plausibility of our modeling framework (Figure 4), As expected at low OD batch cultures, the photosynthetic efficiency of *Synechocystis* obtained by our model simulations showed that efficiency starts to drop the fastest in the initial increase in intensity. Furthermore, owing to the aforementioned dependency of incident light on calibration, this trend appeared accentuated in the calibration cases 2–3 where efficiency turned out to decrease from the highest value, observed at 50 μE where 0.47 g (case 2) and 0.45 g (case 3) of biomass were produced per mole of photons available to the PBR domains, to the lowest value observed at 1,200 μE where 0.12 g (cases 2–3) of biomass were produced per mole of photons. In summary, we provided a quantitative study of light-limited, light-saturated, and light-inhibited growth of the cyanobacterium *Synechocystis* sp. PCC 6803 by monitoring key physiological properties, such as changes in dry weight, gas exchange (O_2_), and photosynthetic efficiency under different lightening and calibration setups in a controlled cultivation environment. The results obtained in this study showed that a quantitative experimental assessment of phototrophic parameters is subject to a number of technical difficulties, which are often reported in insufficient detail. In particular, our analysis illustrated the influence of calibration choices on the characterization of phototrophic growth and activity, which, when superficially treated, can make direct comparison of the literature data difficult and drawn conclusions faulty.

### Light Intensity Profiling Within the Flat-Panel Photobioreactor

**FIGURE 5:**
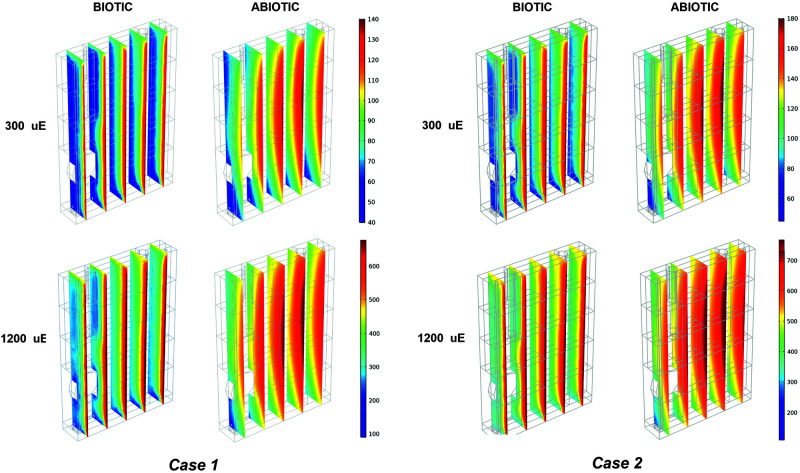
Biotic effects on light transmission through the photobioreactor depend on the incident light and calibration. 3D trend of perceived light intensity along YZ slices of the model PBR acquired at 24 h of simulated time for calibration cases 1–2, in biotic and abiotic conditions, at 300 and 1,200 μE.

The outlined observations and the plausibility shown by our model elicited our interest to exploit our simulation framework to explore the properties of the light that is actually perceived and absorbed by bacterial cells while migrating within the artificially lit vessel of the photobioreactor. The local light intensity profiles were simulated at increasing red-orange light intensities (*I_*s*,in_*), ranging between 50 and 1,200 μE, for each type of calibration of the LED panel. By way of example, Figure 5 and Supplementary Figure 5 show the local light intensity profiles corresponding to *I_*s*,in_* = 300 μE and *I_*s*,in_* = 1,200 μE for the three calibration settings in both biotic and abiotic conditions. Close inspection of our simulation results allowed us to discern fine-grained features of light intensity distribution owing to distinct factors. Sources of variation of the light spatial distribution were identified in the boundary regions, at the interface between the gaseous and liquid phase and at the interface between the liquid phase and the bottom steel base, as well as in the rotating domain created by the stirring bar, as previously detailed in Cordara et al. (2018). Additionally, it is worthwhile noting that these patterns were more accentuated at decreasing values of the incident illumination intensity (Huang et al., 2011). Contrasting the local radiation profiles in various calibration cases allowed us to confirm that the calibration cases 2–3 favor light transmission inside the vessel more than the calibration case 1, at both low and high *I_*s*,in_*, as highlighted in Figure 5 and Supplementary Figure 5.

#### Biotic Effects on Light Transmission in the Photobioreactor Vessel

Contrasting the abiotic and biotic cases allowed us to notice the effects which could be ascribed to the presence of the bacterial culture on light transmission inside the PBR vessel. For each irradiated light and calibration choice analyzed in the biotic cases, light absorption and scattering by bacterial cells accentuated the attenuation of the incident light intensity, when compared to the abiotic cases (Olivieri et al., 2015). In particular, we noticed approximately 6% of light lost in the liquid phase in the abiotic cases against 66% of light lost in the biotic cases, regardless of the calibration setup and of the incident light intensity (Supplementary Table 2). In this regard, the 1D trends of simulated light intensity show a more accentuated light attenuation along the reactor depth, in the biotic case (Figure 3D) compared to the abiotic one (Figure 3C), by way of example, in calibration case 1. Therein, we could notice the highest loss of irradiated light in the inlet and middle area of the vessel (Csögör et al., 1999; Gao et al., 2017a; Naderi et al., 2017). The results of our simulations are in line with the experimentally determined values of light leaving the cultivation system which were previously described in the abiotic (Figure 3A) and biotic (Figure 3B) cases.

As shown in Figure 5 and Supplementary Figure 5, light absorption and scattering by bacterial cells accentuated also the heterogeneity in the light intensity distribution within the liquid phase, when compared to the abiotic case (Soman and Shastri, 2015). These effects get more pronounced as the incident light intensity gets lower. Light reduction is obviously accentuated in the light inlet area of the vessel where, by virtue of reactor design in this study (Supplementary Figure 1), bacterial cells tend to move by effect of the local liquid movement propelled by the stirring bar rotation.

#### Characterization of Light Perception and Absorption by Single Particles

**FIGURE 6:**
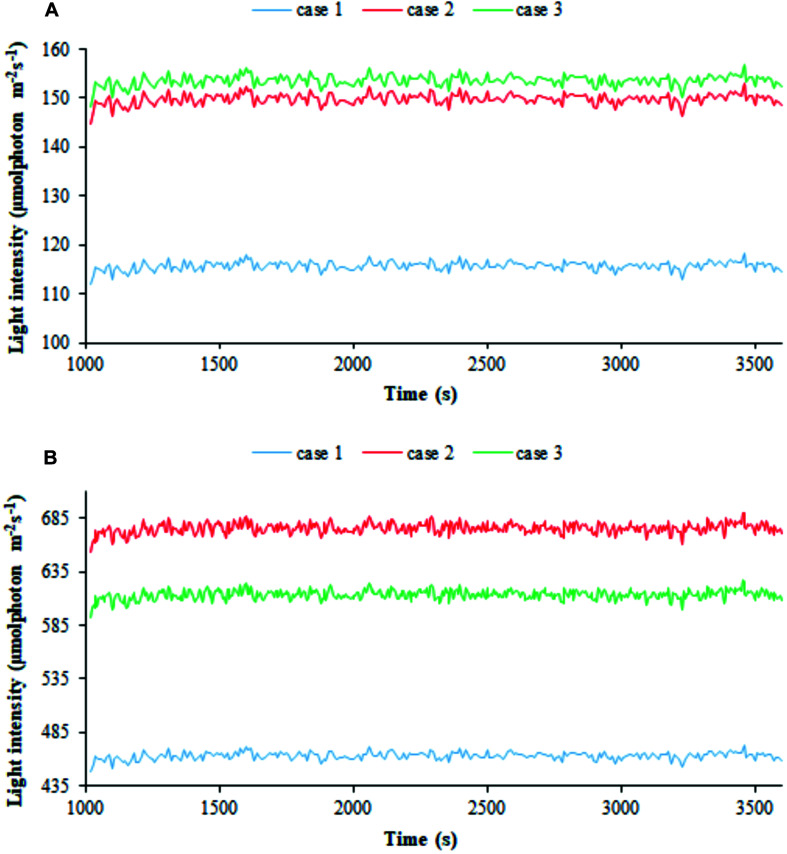
Calibration influences the light perceived by individual cells. 1D simulated trends of radiation perceived by bacterial cells for the three calibration cases at **(A)** 300 μE incident light intensity and **(B)** 1,200 μE incident light intensity. The average of the simulated light intensity values perceived by all the particles is plotted along 1 h of simulated time. Calibration cases are color-coded.

We then employed our modeling framework to relate the trajectories of bacterial cells in the PBR domains featuring different light intensities to cyanobacterial growth. Particle tracing simulations, shown by way of example in Supplementary Figure 6, provide time-dependent trajectories of the light intensity perceived by individual cells and afford the visualization of kinetic and radiation characteristics on particles’ skin (Olivieri et al., 2015; Loomba et al., 2018). We thus computed the one-dimensional trends of the radiation perceived by individual cells along the simulated time (Olivieri et al., 2015; Soman and Shastri, 2015; Iluz and Abu-Ghosh, 2016). The average value of the perceived light intensities over all the cells at each discrete time in the 1 h window of simulated process is displayed in Figure 6 in the three calibration cases at two extreme light intensities, 300 and 1,200 μE. The shown trends, net of fluctuations ranging between 3 and 6% of the average over the whole cells, and 1 h simulated time, confirmed that, at a certain settled *I_*s*,in_* value, the calibration setup influences the light actually perceived by cyanobacterial cells, with the latter being higher in cases 2–3 than in case 1. Moreover, the influence ascribed to calibration became accentuated when lowering the settled light intensity.

**FIGURE 7:**
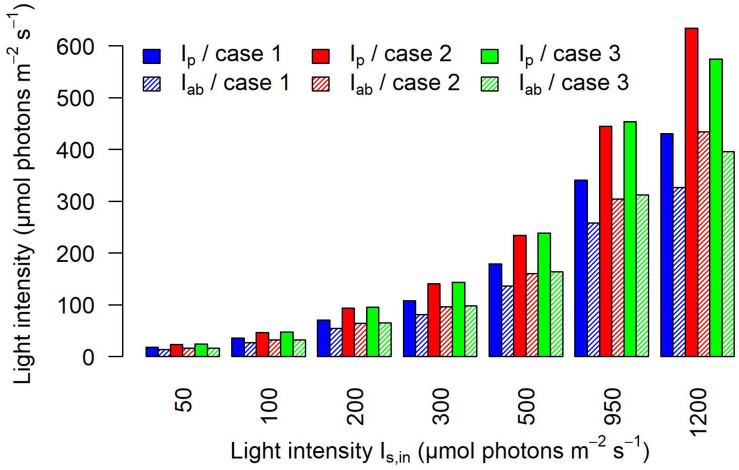
Calibration affects the light perceived and absorbed by individual cells. The barplot shows the light perceived *I*_*p*_ and the light absorbed *I*_*ab*_ on average by cyanobacterial cells at each discrete simulated time, for each calibration case and each incident light intensity *I_*s*,in_*.

Figure 7, which reports the averaged values over the whole cells at a discrete simulated time, confirms that the light intensity that is actually perceived and absorbed by bacterial cells is substantially lower than the light prescribed. The perceived light (*I*_*p*_) is reduced by approximately 64% in calibration case 1, 53% in calibration case 2, and 52% in calibration case 3. When considering the amount of light absorbed by the culture (*I*_*ab*_), our simulation results show that in case 1, the culture absorbs about 27% of the light sent; in case 2, about 32%; and in case 3, about 34% (Supplementary Table 3). As previously noted, the main difference in the light perceived and absorbed is registered between case 1 and cases 2–3, where the trends are similar to each other, for each simulated incident light intensity.

#### Estimation of the Photosynthetic Regime of the Algal Culture by the Simulation Framework

The parameters of the modified Monod kinetics were fitted using a self-implemented Matlab code according to Eq. (33) (del Rio-Chanona et al., 2018). The relevant parameters are *μ_*max*_*, *k_*I*,s_*, and *k*_*I,i*_ which account for the maximum growth rate, light saturation, and photo-inhibition, respectively. These parameters are only dependent on cyanobacterial properties and were fitted from the experimental data on the growth rate previously displayed in Figure 2 and employed to obtain the modeled trends also shown in the same figure. The estimated values for the parameters in the Monod kinetics model are reported in Table 2. The estimated parameters values enabled us to gauge how the different calibration setups of the radiant LED panel influence the photo-limitation regime under low light intensity, the photo-saturation regime under optimal light intensity, and the photo-inhibition regime under intense light intensities (Pilon et al., 2011; Kommareddy and Anderson, 2013). More specifically we could conclude that: (i) case 1 is associated with a higher photo-limitation compared to cases 2–3; (ii) case 1 is associated with a higher photo-saturation compared to cases 2–3; (iii) cases 2–3 are associated with accentuated photo-inhibition compared to case 1.

**TABLE 2:** Comparison of saturation/inhibition/limitation parameters corresponding to each calibration case.

Calibration	*K*_*I,S*_ [W m^–2^]	*K_*I*,i_* [W m^–2^]	μ_*max*_ [h*^–^*^1^]
Case 1	114.5	72.46	0.364
Case 2	43.32	114.9	0.221
Case 3	55.39	82.64	0.265

**μ_*max*_, k_*I,s*_, and k_*I,i*_ account, respectively, for the maximum growth rate, light saturation, and photo-inhibition.**

**FIGURE 8:**
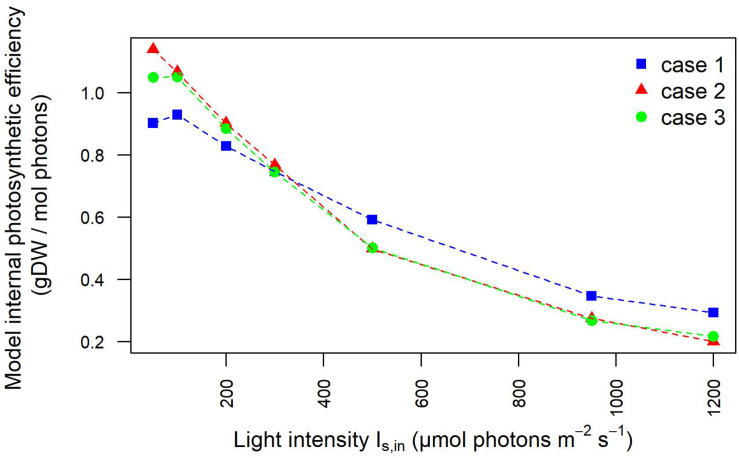
Photosynthetic efficiency based on light intensities at the inlet/outlet of the liquid phase decreases at increasing light intensity. Photosynthetic efficiency is displayed at increasing light intensity under each calibration case. Calibration cases are color-coded. The values shown are derived from simulated light intensities at the inlet and outlet of the liquid phase.

These results are reflected also in the trends of the photosynthetic efficiency displayed in Figure 8. Here, differently from Figure 4, the photosynthetic efficiency has been calculated from the simulated values of the light in input and output of the liquid phase (Supplementary Figure 1), hence referring only to the culture domain, and not to the whole reactor system, as it occurred in relation to Figure 4. As a consequence of it, the photosynthetic efficiency values displayed in Figure 8 are clearly higher than those shown in Figure 4, since the estimates here do not take into account the losses of transmitted radiation due to the other domains of the photobioreactor (Huang et al., 2011). The lower values of the light perceived and absorbed by bacterial cells in case 1 compared to cases 2–3 reflected in the photosynthetic efficiency values: at lower incident light intensity, efficiency is 20% lower for case 1 than for cases 2–3, at high intensities the photosynthetic efficiency is 37% higher in case 1 compared to cases 2–3 (Supplementary Table 4). These findings justify the trends of growth rate and dissolved oxygen shown in Figure 2, further validating the considerations previously proposed on the influence of calibration setups on the characterization of relevant physiological parameters.

#### Light Absorption Coefficient Depends on Bacterial Biomass Concentration

**FIGURE 9:**
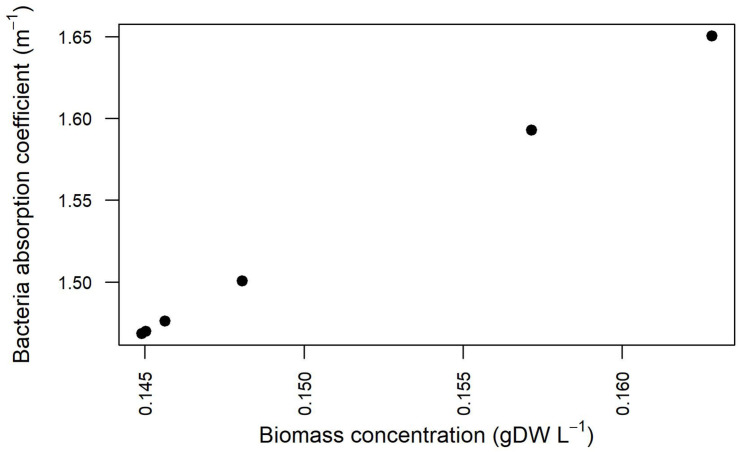
Absorption coefficients for *Synechocystis* at different biomass concentration values for the three calibration cases.

The estimation of the parameters related to the transmission of light in the modeled domain hosting the cell culture was enhanced by explicitly taking into account the experimentally detected light values and the simulation results pertaining cell density at different I_*s*,in_ and growth kinetics (Supplementary Table 5). The relationship between the bacterial absorption parameter and the bacterial biomass concentration resulting from the fitting procedure is shown in Figure 9. The values and the trend obtained from our simulations and shown in Figure 9 are in line with previously published reports according to which the absorption coefficient increases with increasing cell concentration (Agusti and Phlips, 1992; Molina Grima et al., 1994), varying from around 1.34 m^–1^ for 0.1 gDCW/L to around 4 m^–1^ for 2 gDCW/L (Zhang et al., 2015).

## Discussion

Optimal exploitation of the production capabilities of cyanobacterial growth potential is reliant on how the inherent properties of photobioreactors can be adjusted to create an environment able to accommodate the culture growth and physiology optimally. The photophysiological performances, which are usually detected experimentally, result from the superposition of several highly variable aspects related to gas exchange, mixing regime, reactor geometry, and light intensity distribution (Béchet et al., 2013; Huang et al., 2017). Direct access to the processes within the reactor environment is burdensome; therefore, simulation modeling is invaluable to shed insights into the reasons underlying the photosynthetic performances (Wang B. et al., 2020). The engineering and/or operational solutions consequently devised can improve biomass photosynthetic growth efficiency and productivity. For these reasons, we coupled the acquisition of experimental data from a carefully designed campaign to a mechanistic simulation modeling of a wide range of processes intervening in a conventional flat-panel photobioreactor in order to provide a quantitative study of light-limited, light-saturated, and light-inhibited growth of *Synechocystis* sp. PCC 6803 turbidostat cultures.

Robust quantification of light intensity dependence of cyanobacterial activity (Sarkar et al., 2019; Zavřel et al., 2019; Toyoshima et al., 2020) is instrumental for predicting a bioprocess performance and designing an efficient photobioreactor (Zhang et al., 2015; Papacek et al., 2018). Cyanobacterial physiology at different light regimes was thus characterized by gauging the oxygen evolution (Jeon et al., 2005; Cordara et al., 2018) and by expressing the photosynthetic efficiency as moles of photons absorbed per gram of biomass formed (Schuurmans et al., 2015). Our characterization of cyanobacterial response to increasing incident light intensities allowed us to identify a range of low incident light intensities, which afford fast cellular growth and pronounced release of oxygen in the medium, and a range of high light intensities inducing photoinhibition (Ogawa et al., 2018). Photosynthetic efficiency estimates were found in agreement with the observed oxygen evolution. The fast decrease of photosynthetic activity at moderately increased light intensity is in line with experimental data acquired also in previous studies, if we account for the difference existing in growth rate and light transmission (Schuurmans et al., 2015). Notably, we could observe that the highlighted trends were sensibly affected by the calibration choice.

The experimentally observed relation between the physiological properties and the supply of external light was effectively recapitulated, corroborating the validity of our modeling framework. Combining the simulation of the evolution of cells with the reconstruction of differently lit domains of the photobioreactor allowed us to relate the observed photophysiological properties to the transmission of light in the medium and to the patterns of light actually perceived and absorbed by bacterial cells. The acquisition of a deeper understanding of the relation between physiological traits and local patterns in light transfer can inform the photobioreactor design and the operational choices (Gao et al., 2018). Leveraging experimental data on light intensity and simulated data on cell density and growth kinetics, our modeling approach also allowed us to explore the dependence of light absorption on cell concentration (Huang et al., 2012). The latter reparameterization is useful to enhance our understanding of light distribution and, hence, to guide the optimization of the photobioreactor operating conditions (Huang et al., 2017).

Of particular interest were the trends of physiological parameters (growth rate, dissolved oxygen, and photosynthetic efficiency) at increasing light intensity with respect to different calibration cases. Indeed, we showed how the assessment of the physiological response to illumination depends on the initialization of the externally supplied light with regard not only to prearranged light wavelength and intensity but also to the specific light calibration procedure. Our simulation results demonstrated that setting the model with the light resulting from different calibrating configurations impacts differently on the local light distribution inside the photobioreactor and thus on the light actually perceived by the bacteria, obviously influencing microorganism growth kinetics.

In summary, our modeling framework can be integrated into procedures for effective and stable control based on the monitoring of process data that cannot be directly measured and for dynamic bioprocess modeling. The combination of experimental data acquisition and simulation modeling allows understanding how experimental data, often partially overlooked, have to be included to enhance the accuracy and extrapolation capabilities of the model to shorten the bioprocess development. Finally, our study suggests that full transparency in reporting experimental design and methodological details is paramount to reproduce and understand scientific outcomes and build upon valuable findings to foster the collective achievement of innovation goals in bioprocess engineering.

## Data Availability Statement

The original contributions presented in the study are included in the article/Supplementary Material, further inquiries can be directed to the corresponding author/s.

## Author Contributions

NV, AC, and AR: conceptualization. NV: modeling methodology. NV and AR: formal analysis and writing—original draft. AC and GU: experiments. AC: experimental data treatment. NV, AC, GU, and AR: writing—review and editing. All authors contributed to the article and approved the submitted version.

## Conflict of Interest

The authors declare that the research was conducted in the absence of any commercial or financial relationships that could be construed as a potential conflict of interest.

## Supplementary Material

Supplementary Figure 1 **(A)** Body of the flat panel PBR FMT150.2/400 composed of a 390 ml transparent removable flat vessel. On top of the vessel, a stainless lid accommodates different tubes, connectors, and sensors. The base of the instrument contains a control unit with analogic and digital electronic circuits. Enlarged the details of the red and blue LEDs installed in the light panel of the reactor, the densitometer, and the fluorometer. **(B)** Red and blue LED spectra of PBR FMT150.2/400. **(C)** Transmission spectrum of cyanobacterial culture affected by light absorptions, light scattering. The lines and arrows indicate wavelength of the light sources present in the flat panel reactor and the detection range of the detector filter. **(D)** 3D modeled geometry of PBR with modeled domains selection: 1-closing, 2-inoculum, 3-sparger, 4-liquid immission, 5-sampling/liquid extraction, 6-culture, 7-stirring bar domain, 8-stainless steel walls of vessel, 9-base of vessel, 10-gas outlet, 11,O2 probe, 12-pH probe, 13-air, 14-glass walls of vessel, 15-LED panel.

Supplementary Figure 2 LED panel calibration trendlines in the three calibration cases.

Supplementary Figure 3 Stability of growth rate during 24 h at each step-wise increase in the intensity value *I_*s*,in_* supplied to the PBR. By way of example, the figure refers to the calibration case 1.

Supplementary Figure 4 PBR design. **(A)** 3D CAD geometries and **(B)** system mesh.

Supplementary Figure 5 3D trend of perceived light intensity along YZ slices of the model PBR for calibration case 3, in biotic and abiotic conditions, at 300 and 1,200 μE.

Supplementary Figure 6 3D, lateral (XZ), and top (XY) view particles spatial distribution in function of skin velocity **(A)** and radiation perceived **(B)**.

Supplementary Table 1 Model Equations. The table displays the equation sets employed to simulate each phenomenon included in the model. The table reports also the domains in the 3D geometry of the photobioreactor to which each listed equation applies.

Supplementary Table 2 Experimental and simulated light intensities at the outlet of the PBR in abiotic and biotic configurations at increasing incident light intensity.

Supplementary Table 3 Simulated values for the average light perceived and absorbed by bacteria at all the simulated *I_*s*,n._*

Supplementary Table 4 Comparisons of efficiency of photosynthesis between the three calibration cases at increasing incident light intensity.

Supplementary Table 5 Model parameters for heat transfer with radiation.

## Nomenclature

**Table:**

Symbol	Description	Symbol	Description
*A*	area, m^2^	*v_F*	the convective velocity, m s^–1^
c_*d*_	mass fraction of dispersed phase, kg kg ^–1^	*w*	volume fraction
*C*	concentration, mol m^–3^	*X*	mass fraction
*C*_*p*_	specific heat at constant pressure, J m^–3^ K^–1^	I_*s,in*_	Incident light intensity set via software, W m^–2^
*D*	diffusion coefficients, m^2^ s^–1^	*I*_*p*_	Light intensity perceived by bacterial cell, W m^–2^
*D*_*md*_	turbulent dispersion coefficient, m^2^ s^–1^	*I*_*ab*_	Light intensity absorbed by bacterial cell, W m^–2^
*e*	enthalpy flux density, J m^–2^ s^–1^	*_*FD*_*	Drag force
*E*_*A*_	activation energy, J mol^–1^	_*Fg*_	Gravity force
*F*	force term, kg m^–2^ s^–2^	*_*Fext*_*	Additional force
*G*	incident light radiation, W m^–2^	*_*mp*_*	Particle mass
*h_*j*_(T)*	enthalpies heat flux densities, J m^–2^ s^–1^	*_*rhop*_*	Particle density
*I*	incident light intensity, W m^–2^	*_*dp*_*	Particle diameter
*I*_*b*_	black body radiation, Wm ^–2^	*_*xp*_*	Particle trajectory
*J*	diffusion vector	*_*vp*_*	Particle velocity
k	turbulent kinetic energy, m^2^s^–3^	**Greek symbols**	
*K*_*r*_	reaction rate constant, m^2^ s^–1^	β	extinction coefficient, m^–1^
*m*	mass of species, kg	ε	turbulent energy dissipation, m^2^s ^–3^
*m*_*dc*_	mass transfer from dispersed to continuous phase, kg m ^–3^s ^–1^	*k*_*c*_	effective thermal conductivity coefficient, W m^–1^ K^–1^
*M*	molar mass, kg mol^–1^	κ	absorbance coefficient, m^–1^
*n*	flux density, mol m^–2^ s^–1^	*μ*	dynamic viscosity, kg_*f*_ s m^–2^
*n_d*	relative mass flux, mol m^–2^ s^–1^	*μ_*gr*_*	growth rate, h ^–1^
*p*	pressure, Pa	*μ_*T*_*	turbulent viscosity,
*q*	heat flux densities, W m^–2^	*ν*	stoichiometric coefficients
*Q*	volumetric charge density, C m^–3^	*ρ*	density, Kg m^–3^
*Q*_*r*_	radiative flux, W m^–2^	*σ_*S*_*	scattering coefficient, m^–1^
*R*	universal gas constant, J K^–1^ mol^–1^	*τ*	turbulent stress,
*T*	temperature, K	*φ_χ_*	continuous phase fraction, −
*u*	velocity vector, m s^–1^	*φ_δ_*	dispersed phase fraction, −
*u*_*c*_	continuous phase velocity vector, m s^–1^	*ω*	rotational velocity, rad s^–1^
*u*_*d*_	dispersed phase velocity vector, m s^–1^	η	dynamic viscosity, Pa s^–1^
*u*_*slip*_	slip velocity vector, m s^–1^		
