# Supplementary figures and images for: Computational Analysis of Dynamic Light Exposure of Unicellular Algal Cells in a Flat-Panel Photobioreactor to Support Light-Induced CO2 Bioprocess Development
Source: Front Microbiol. 2021 Apr 1;12:639482. doi: 10.3389/fmicb.2021.639482 (PMC8049116; doi:10.3389/fmicb.2021.639482)

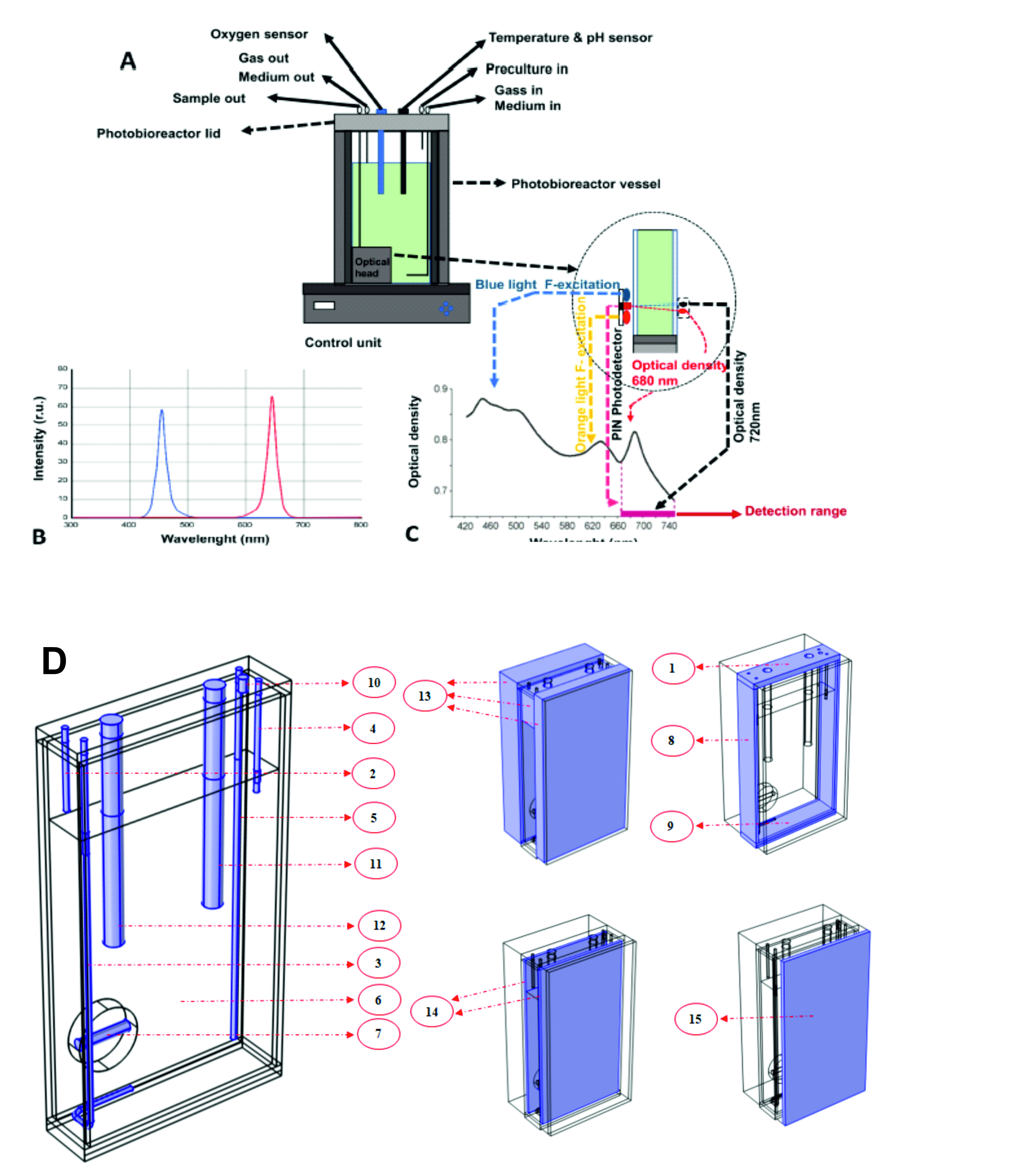

Supplement: Supplementary Figure 1 — (A) Body of the flat panel PBR FMT150.2/400 composed of a 390 ml transparent removable flat vessel. On top of the vessel, a stainless lid accommodates different tubes, connectors, and sensors. The base of the instrument contains a control unit with analogic and digital electronic circuits. Enlarged the details of the red and blue LEDs installed in the light panel of the reactor, the densitometer, and the fluorometer. (B) Red and blue LED spectra of PBR FMT150.2/400. (C) Transmission spectrum of cyanobacterial culture affected by light absorptions, light scattering. The lines and arrows indicate wavelength of the light sources present in the flat panel reactor and the detection range of the detector filter. (D) 3D modeled geometry of PBR with modeled domains selection: 1-closing, 2-inoculum, 3-sparger, 4-liquid immission, 5-sampling/liquid extraction, 6-culture, 7-stirring bar domain, 8-stainless steel walls of vessel, 9-base of vessel, 10-gas outlet, 11,O2 probe, 12-pH probe, 13-air, 14-glass walls of vessel, 15-LED panel. [file Image_1.JPEG]

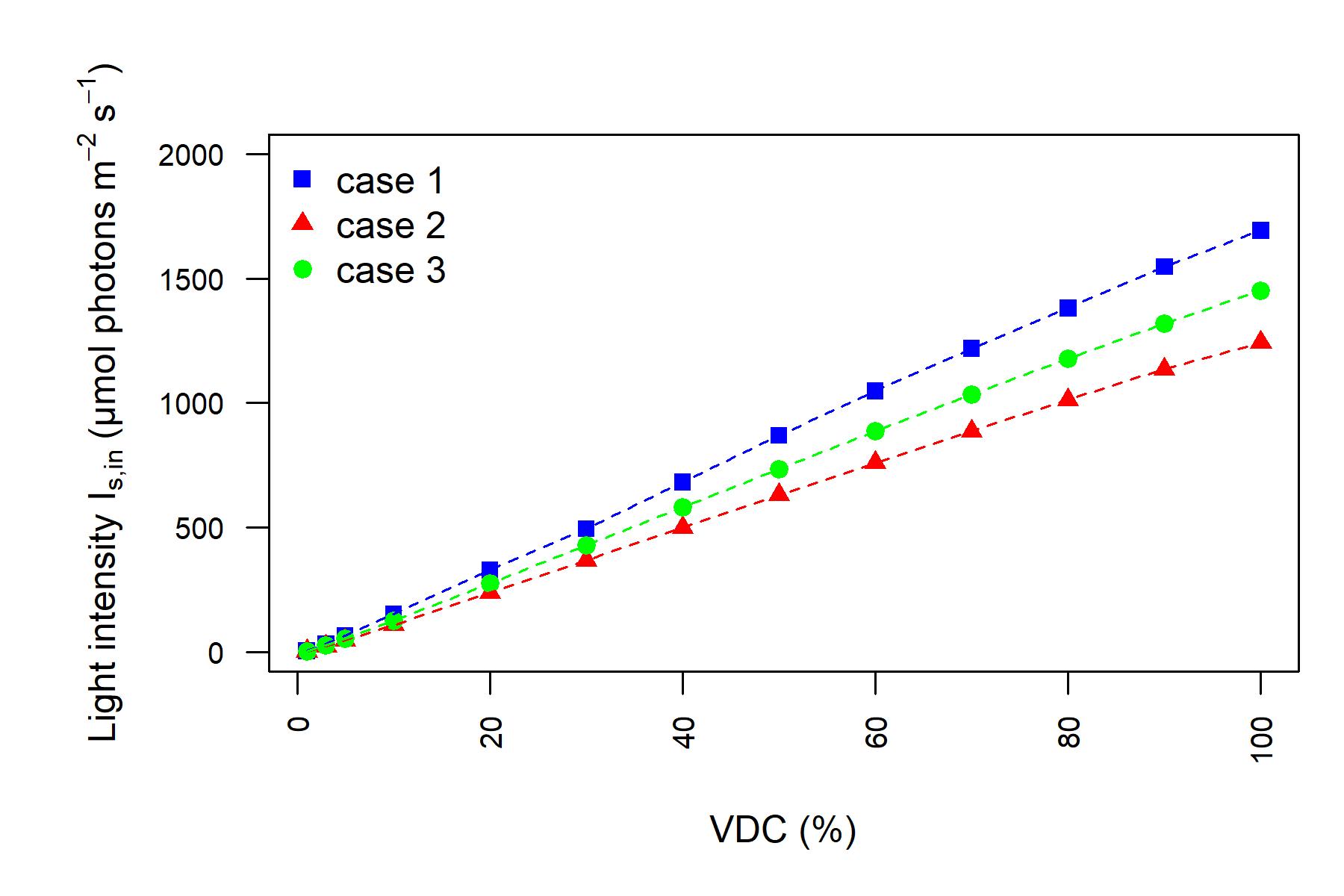

Supplement: Supplementary Figure 2 — LED panel calibration trendlines in the three calibration cases. [file Image_2.JPEG]

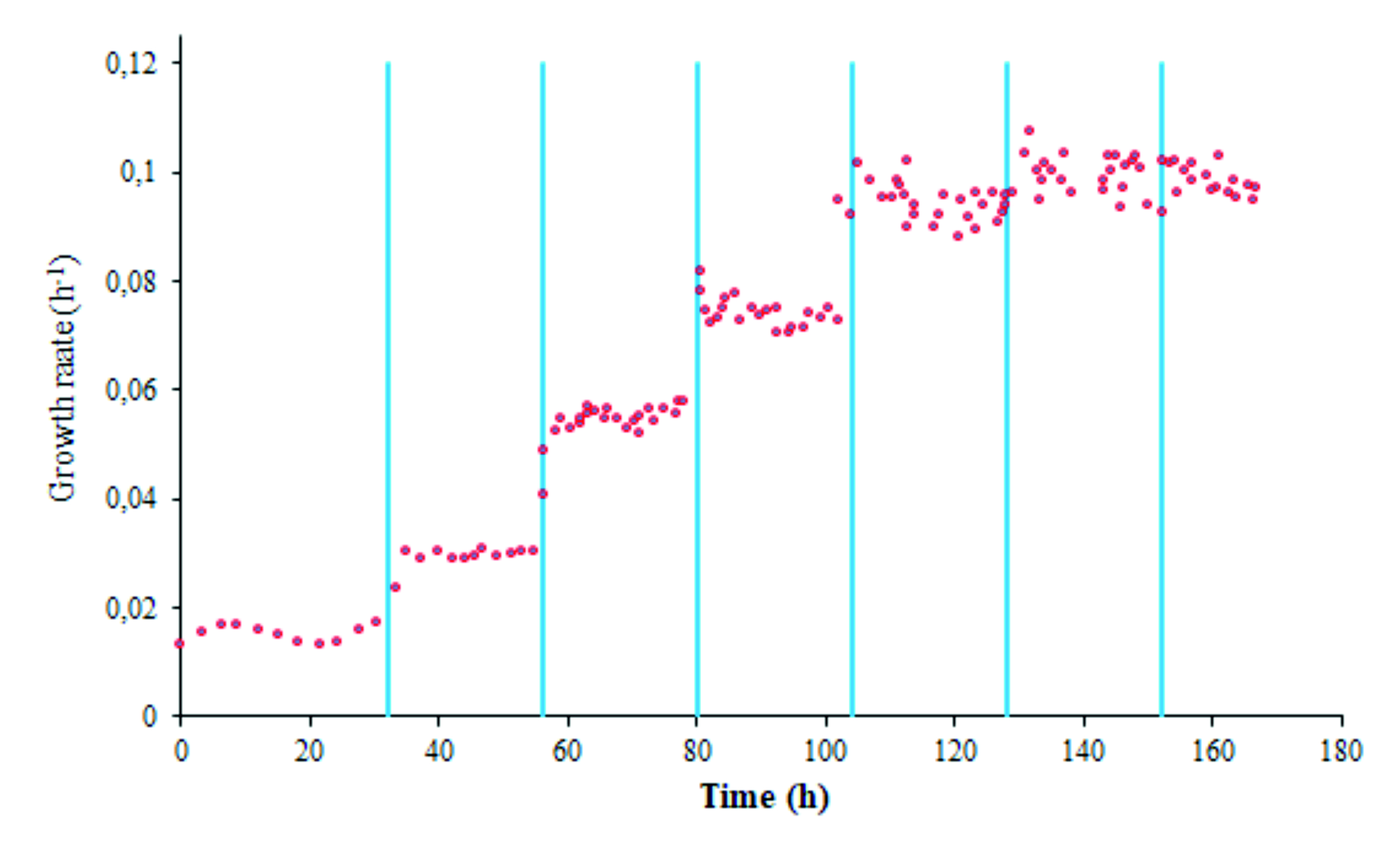

Supplement: Supplementary Figure 3 — Stability of growth rate during 24 h at each step-wise increase in the intensity value Is,in supplied to the PBR. By way of example, the figure refers to the calibration case 1. [file Image_3.JPEG]

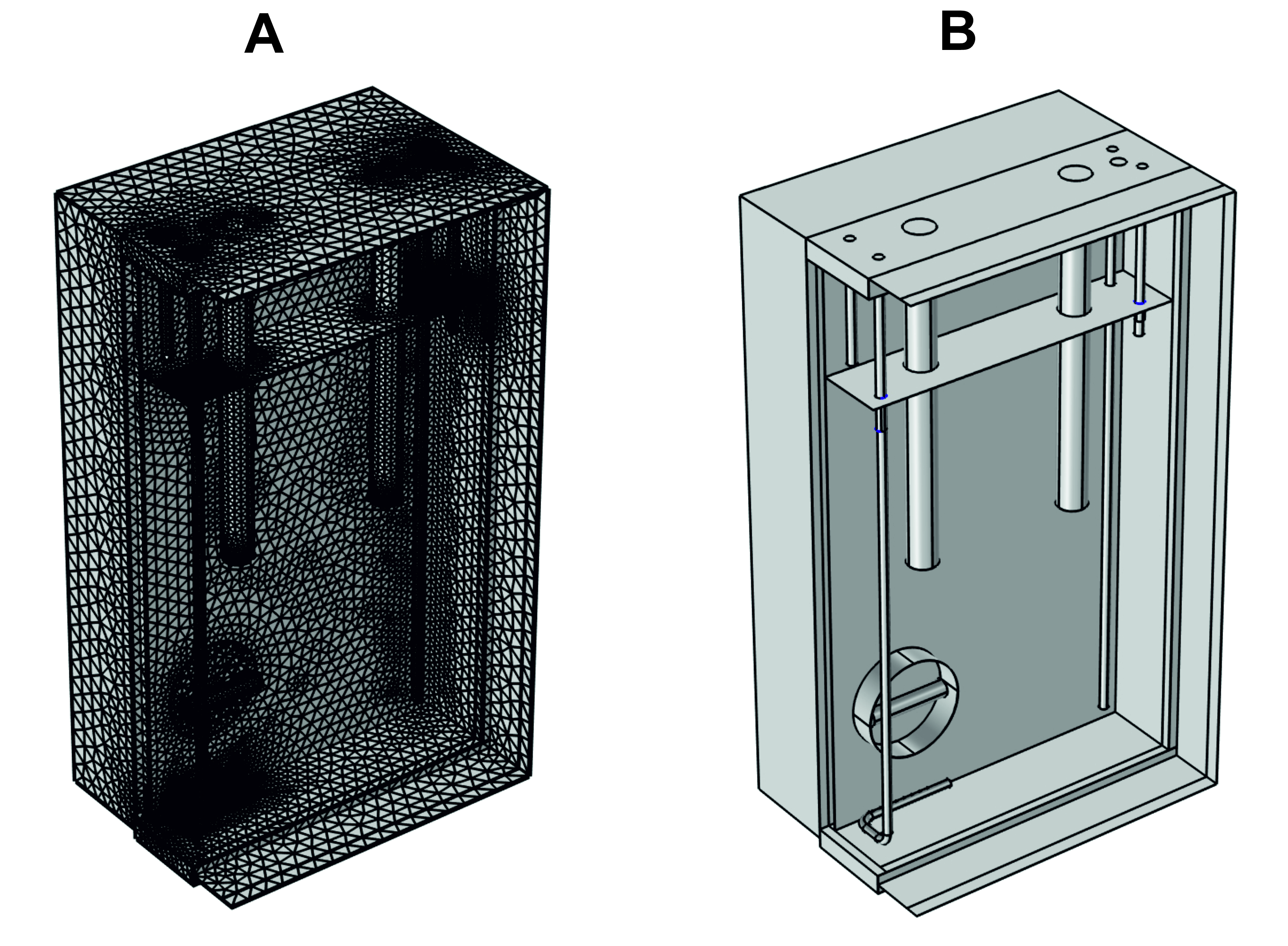

Supplement: Supplementary Figure 4 — PBR design. (A) 3D CAD geometries and (B) system mesh. [file Image_4.JPEG]

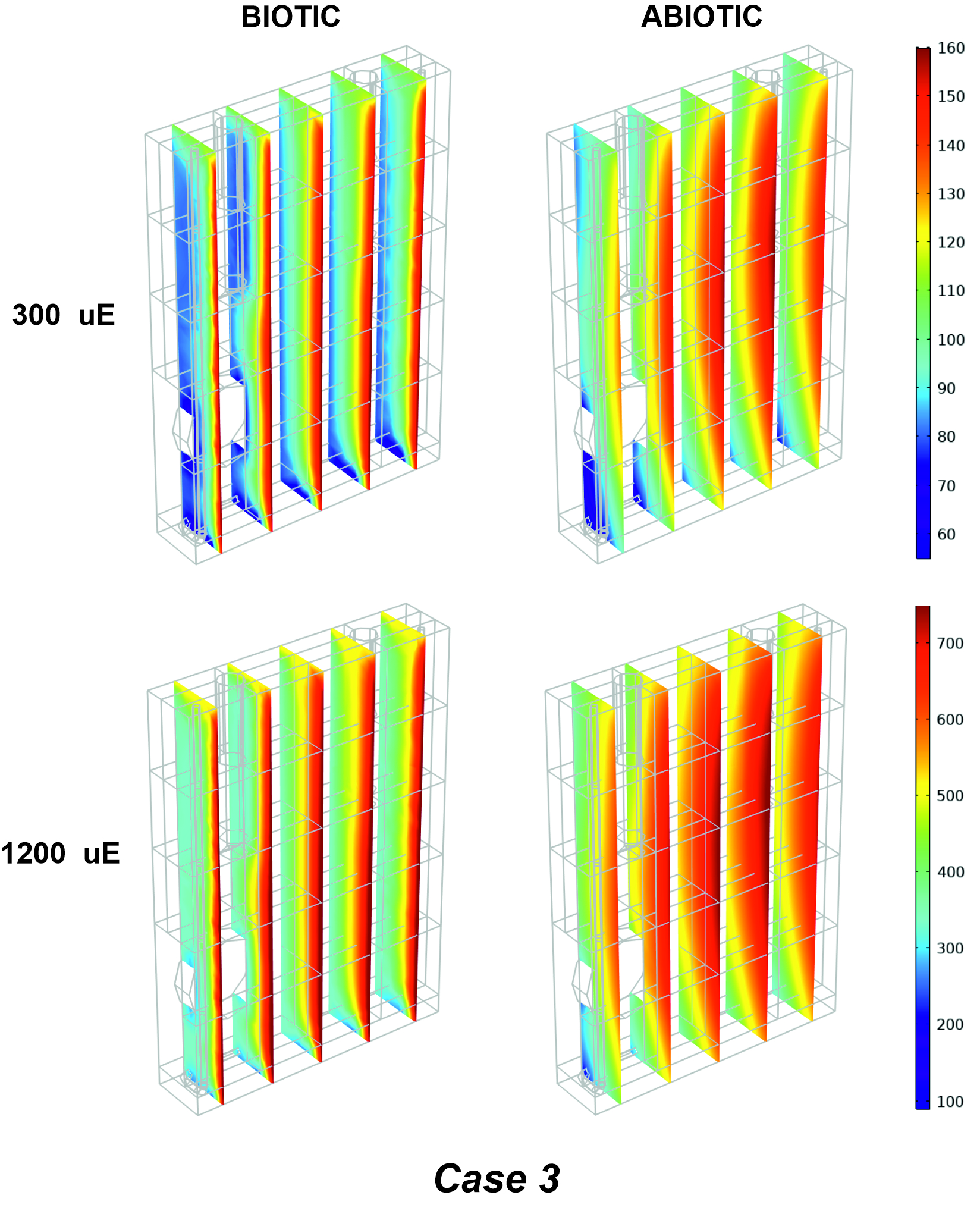

Supplement: Supplementary Figure 5 — 3D trend of perceived light intensity along YZ slices of the model PBR for calibration case 3, in biotic and abiotic conditions, at 300 and 1,200 μE. [file Image_5.JPEG]

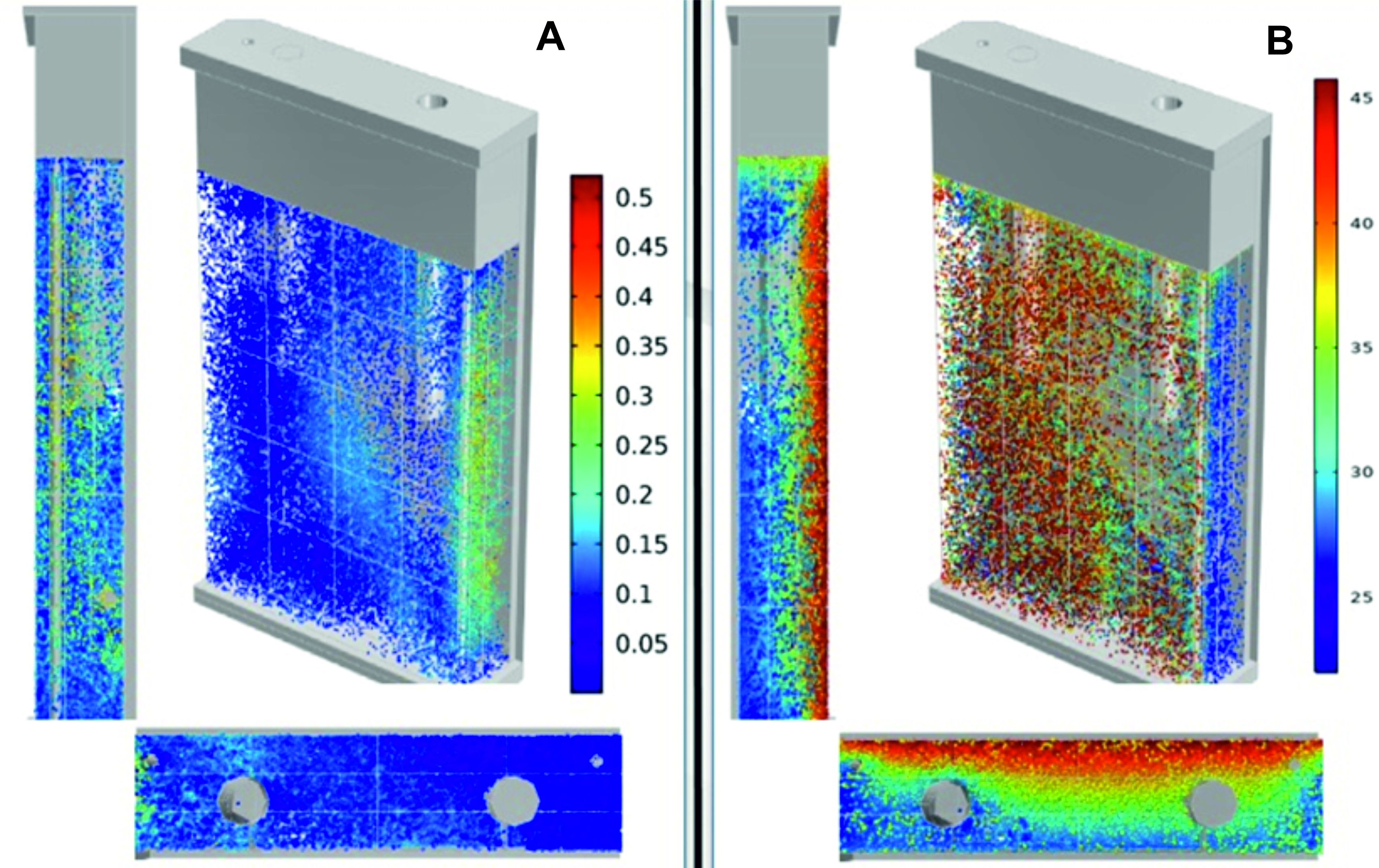

Supplement: Supplementary Figure 6 — 3D, lateral (XZ), and top (XY) view particles spatial distribution in function of skin velocity (A) and radiation perceived (B). [file Image_6.JPEG]
