# Supplementary material for: Computational Analysis of Dynamic Light Exposure of Unicellular Algal Cells in a Flat-Panel Photobioreactor to Support Light-Induced CO2 Bioprocess Development
Source: Front Microbiol. 2021 Apr 1;12:639482. doi: 10.3389/fmicb.2021.639482 (PMC8049116; doi:10.3389/fmicb.2021.639482)
Supplement: Supplementary Table 1 — Model Equations. The table displays the equation sets employed to simulate each phenomenon included in the model. The table reports also the domains in the 3D geometry of the photobioreactor to which each listed equation applies. [file Table_1.docx]

**Supplementary Table 1 Model equations**. The table displays the equation sets employed to simulate each phenomenon included in the model. The table reports also the domains in the 3D geometry of the photobioreactor to which each listed equation applies.

| **Equation** | **Domain** | **Equation number** | **Mathematical model** |
| --- | --- | --- | --- |
| _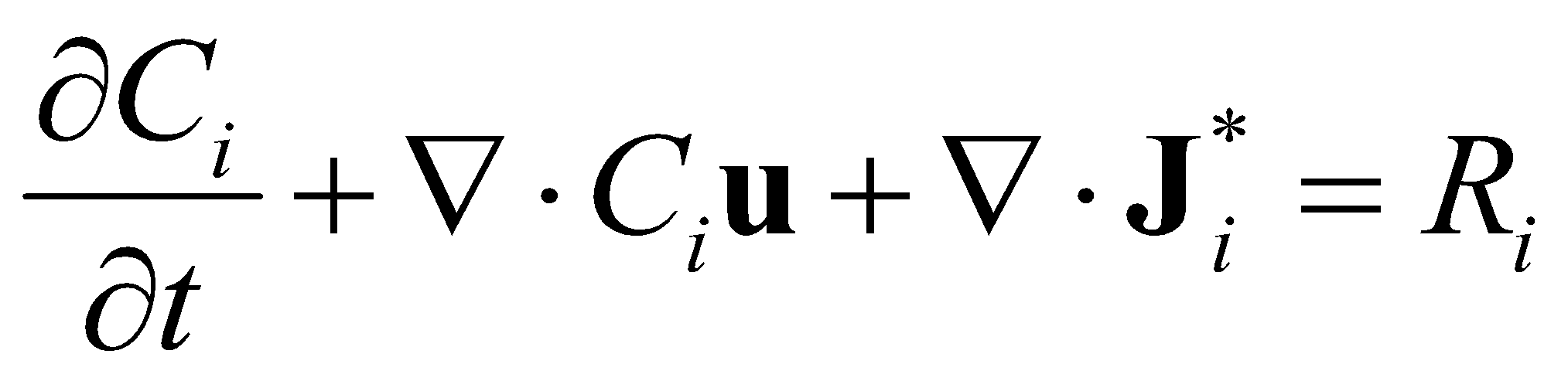_ | 3-4-6-7 | 1 | MASS TRANSFER |
| _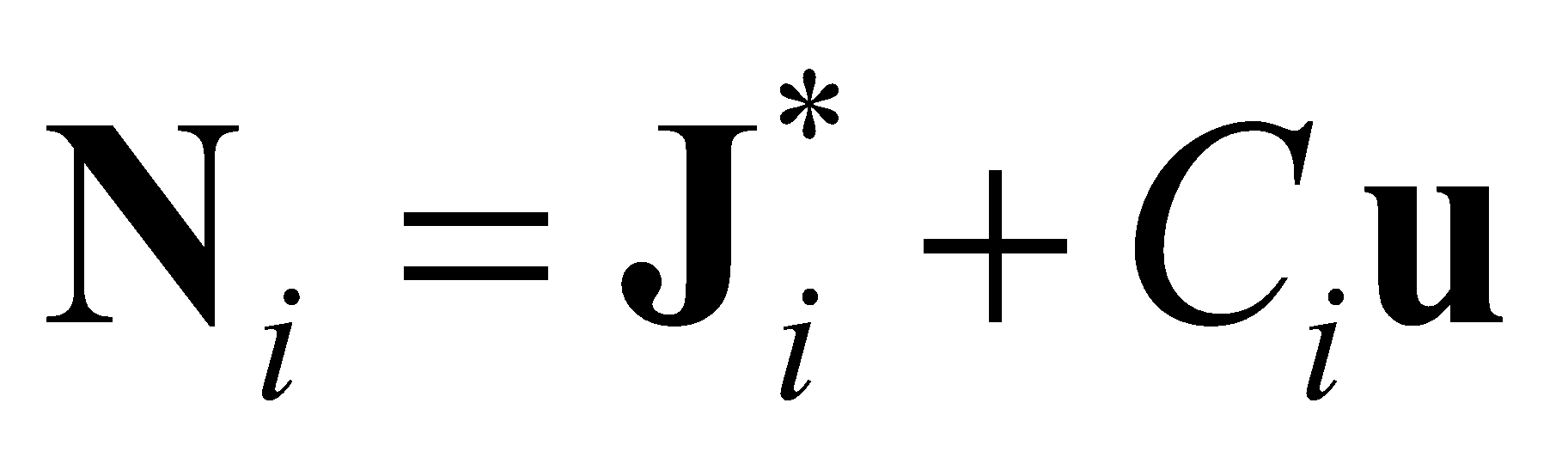_ | 3-4-6-7 | 2 |  |
| _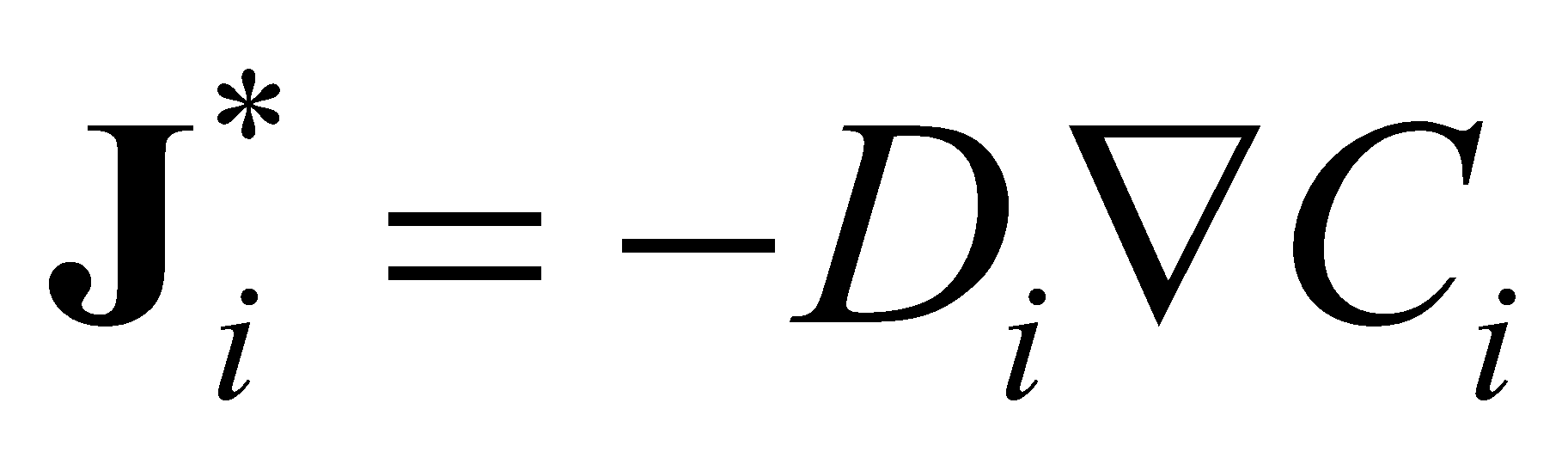_ | 3-4-6-7 | 3 |  |
| _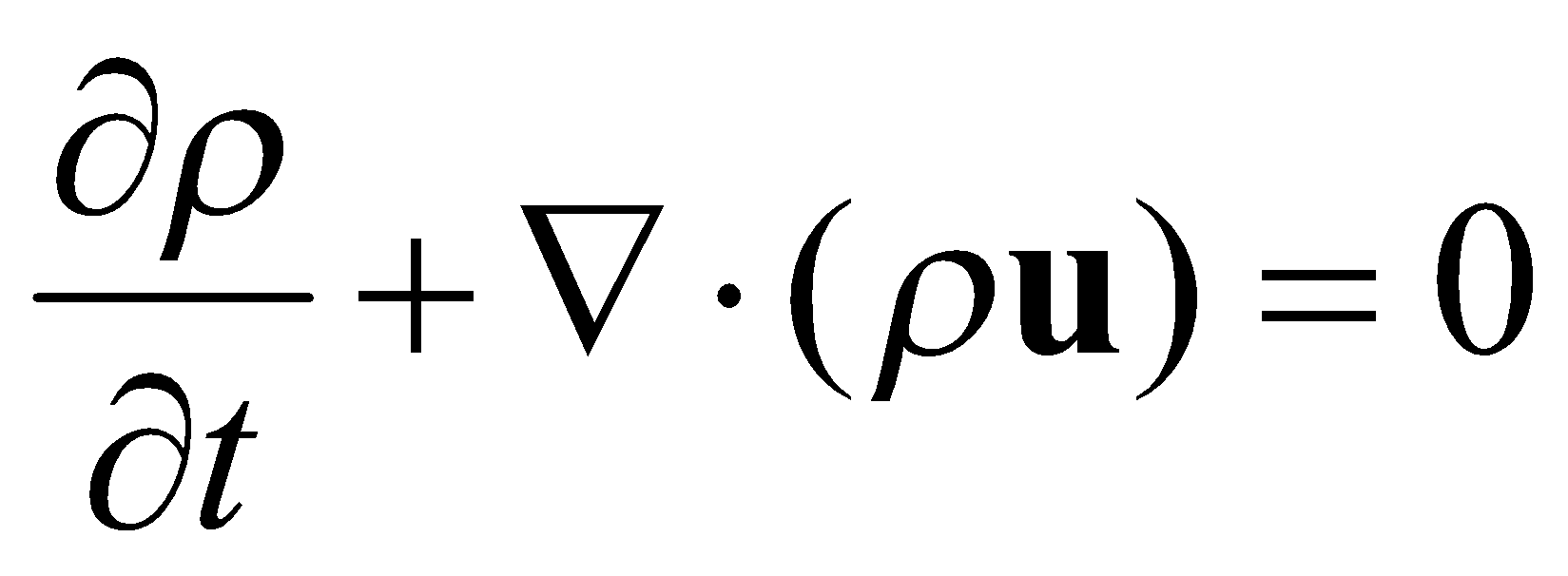_ | 3-4-6-7-10-13 | 4 | FLUID-DYNAMICS |
| _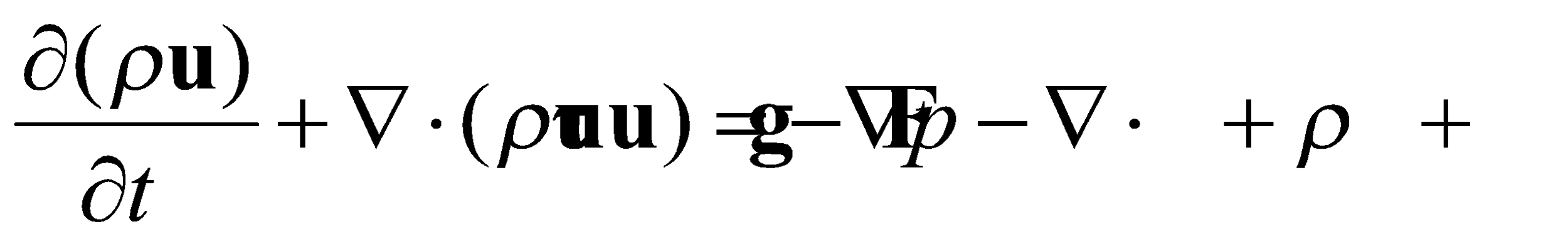_ | 3-4-6-7-10-13 | 5 |  |
| _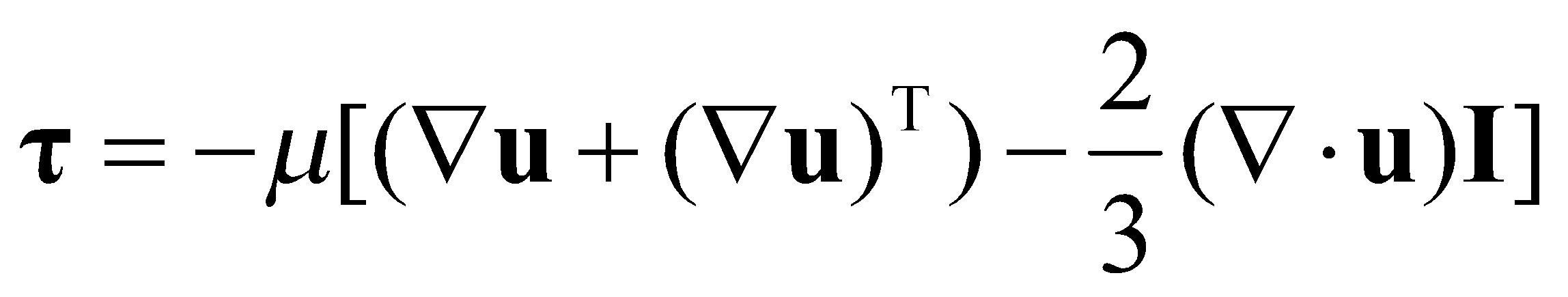_ | 3-4-6-7-10-13 | 6 |  |
| _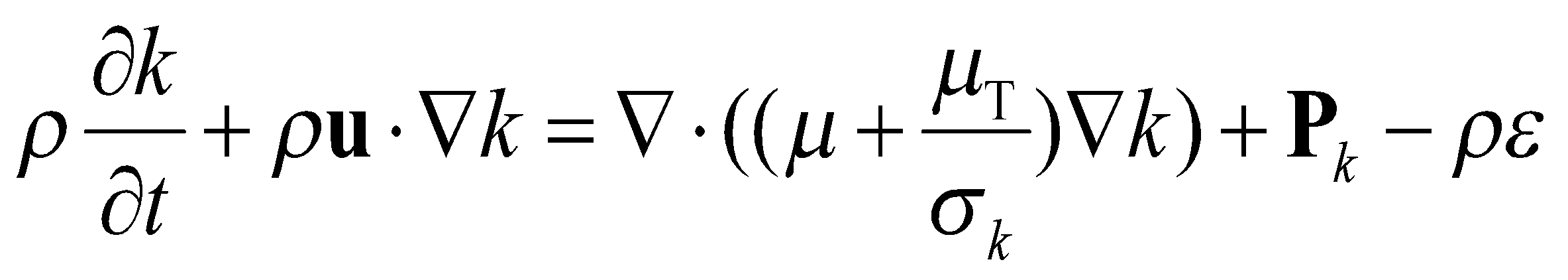_ | 4-6-7 | 7 |  |
| _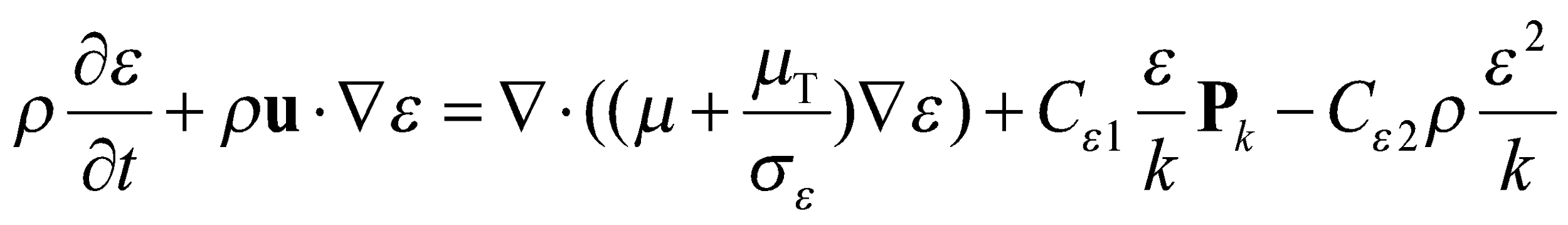_ | 4-6-7 | 8 |  |
| _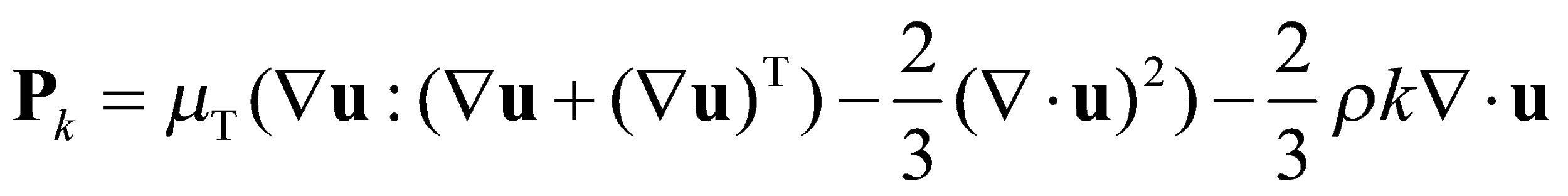_ | 4-6-7 | 9 |  |
| _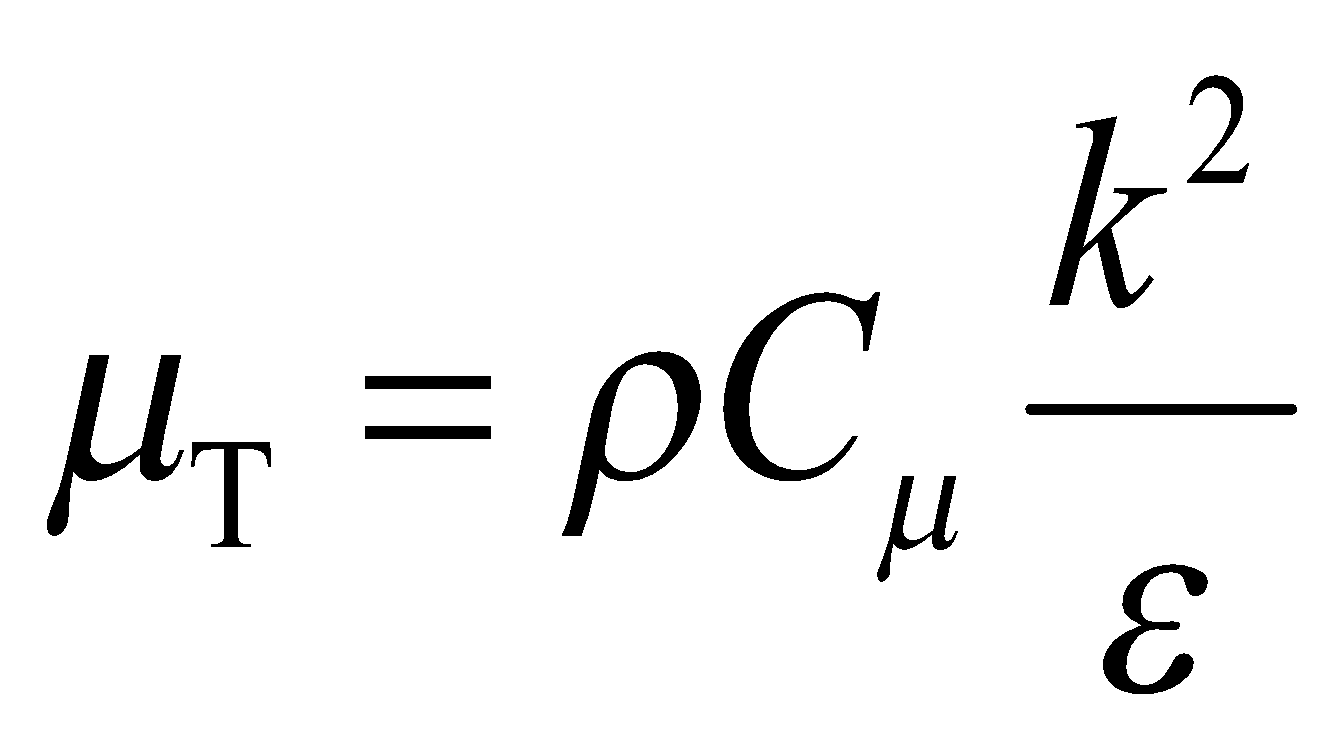_ | 4-6-7 | 10 |  |
| _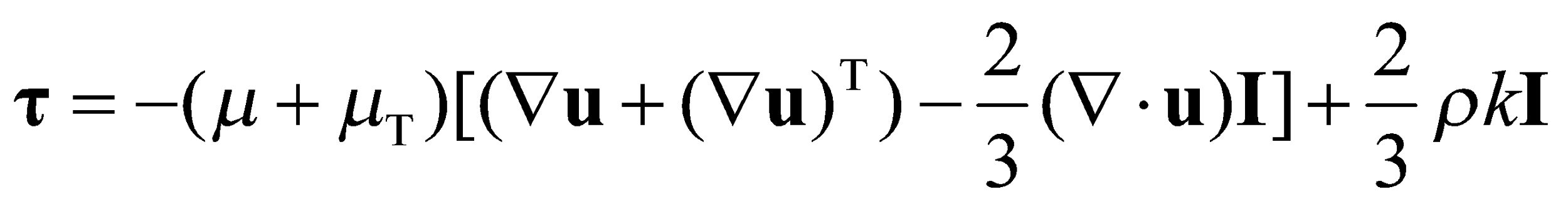_ | 4-6-7 | 11 |  |
| $\emptyset_{c}+ \emptyset_{d}=1$ | 3-4-6-7-10-13 | 12 |  |
| _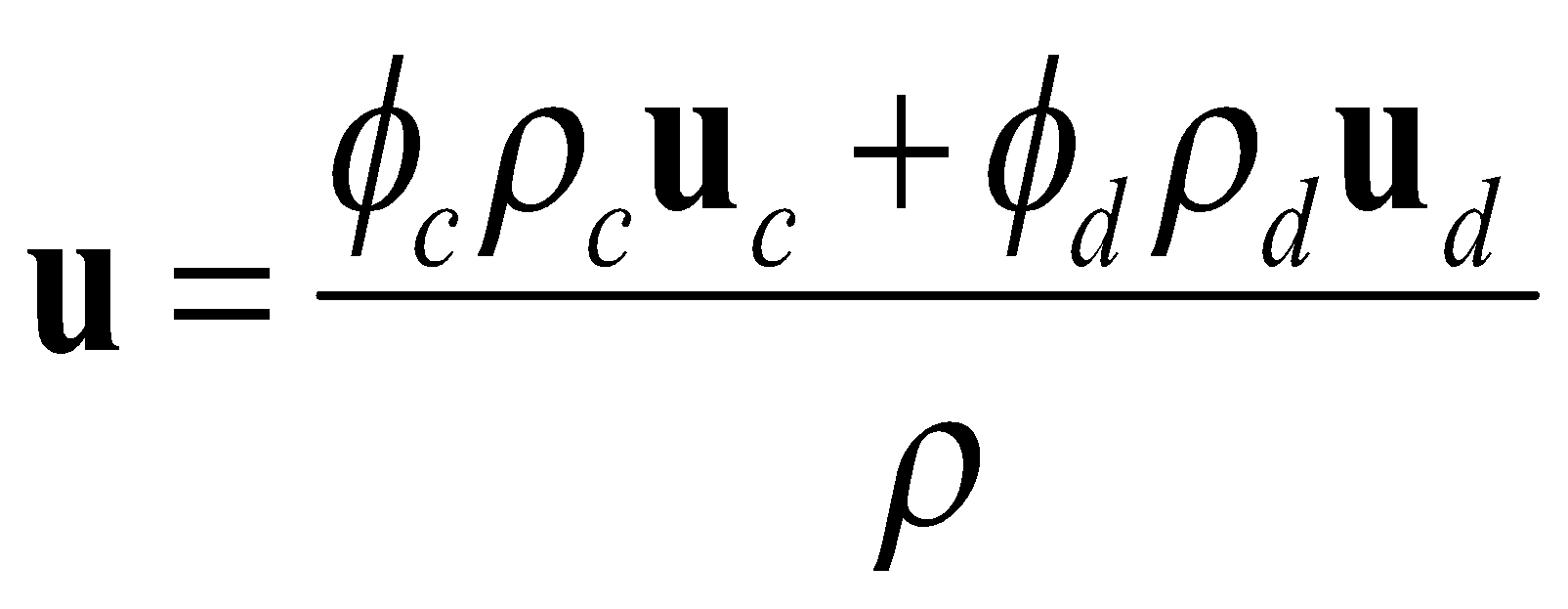_ | 3-4-6-7-10-13 | 13 |  |
| _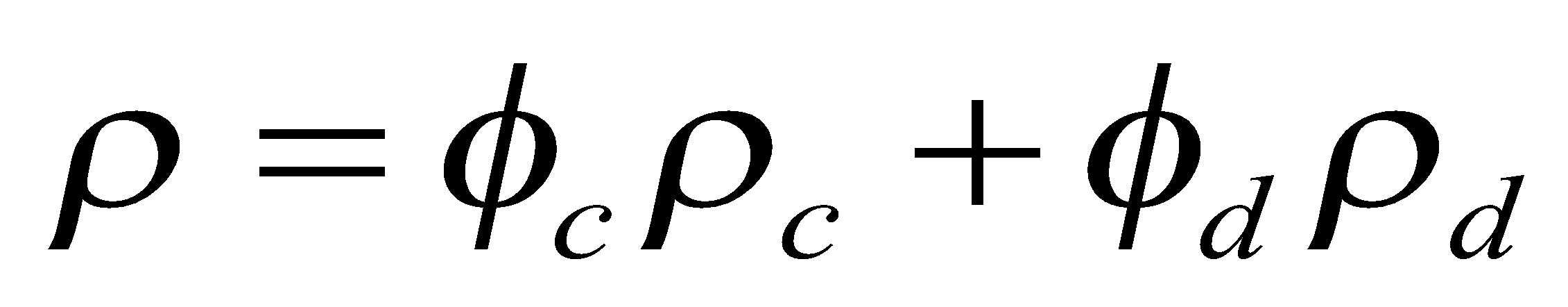_ | 3-4-6-7-10-13 | 14 |  |
| $\mu_{eff}=\mu_{i}+\mu_{i,T}$ | 3-4-6-7-10-13 | 15 |  |
| _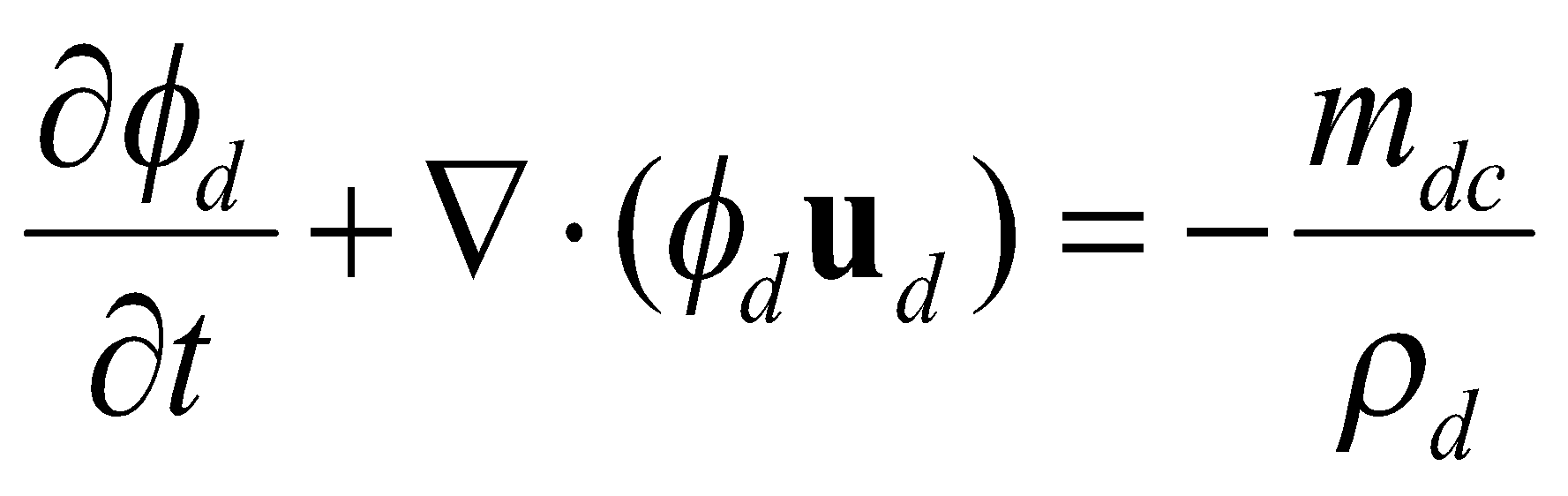_ | 3-4-6-7-10-13 | 16 |  |
| _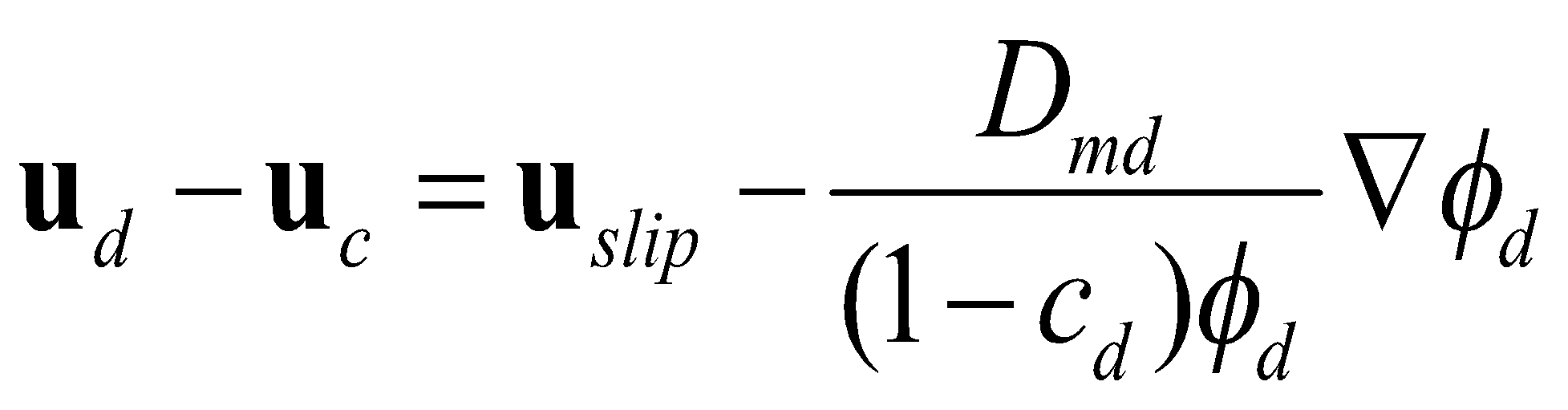_ | 3-4-6-7-10-13 | 17 |  |
| _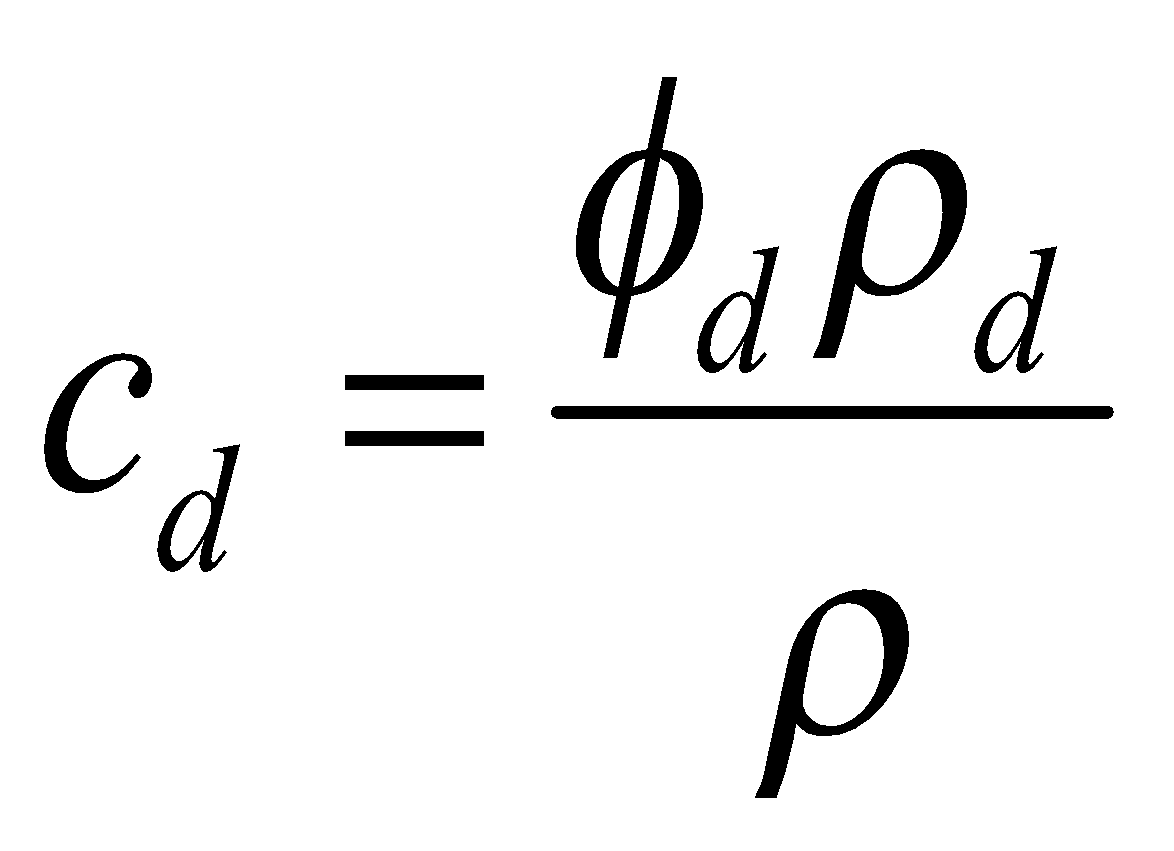_ | 3-4-6-7-10-13 | 18 |  |
| _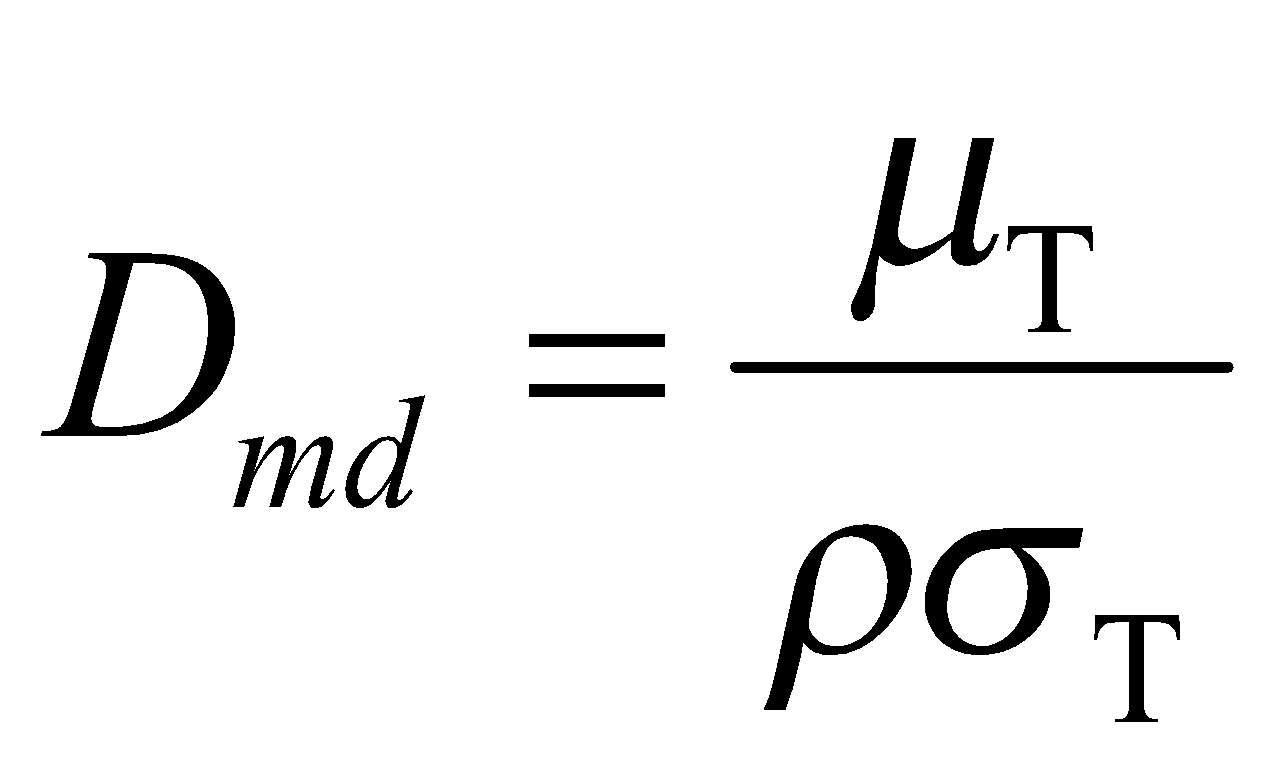_ | 3-4-6-7-10-13 | 19 |  |
| _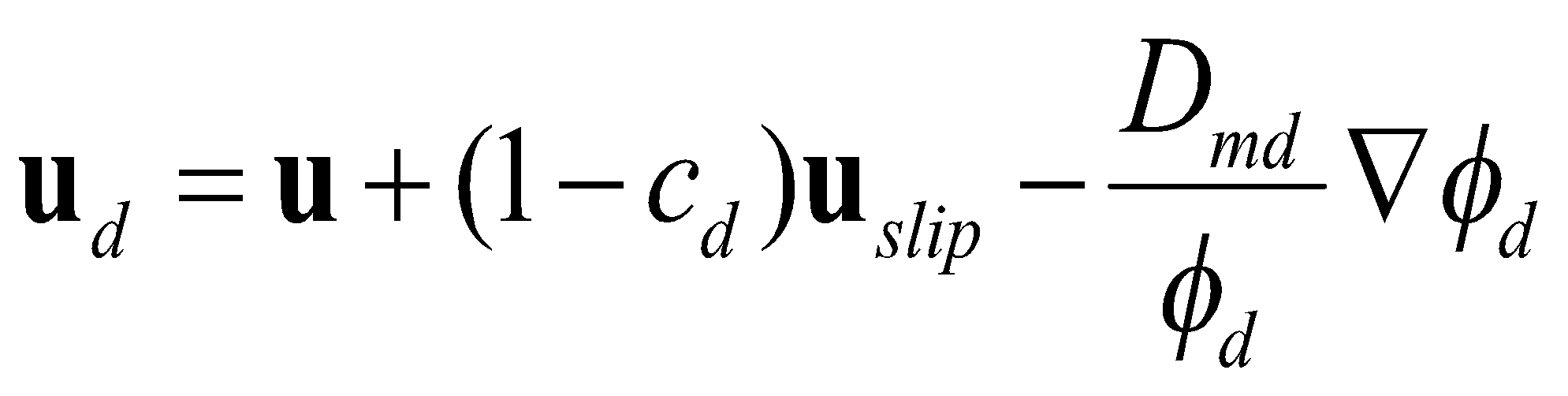_ | 3-4-6-7-10-13 | 20 |  |
| _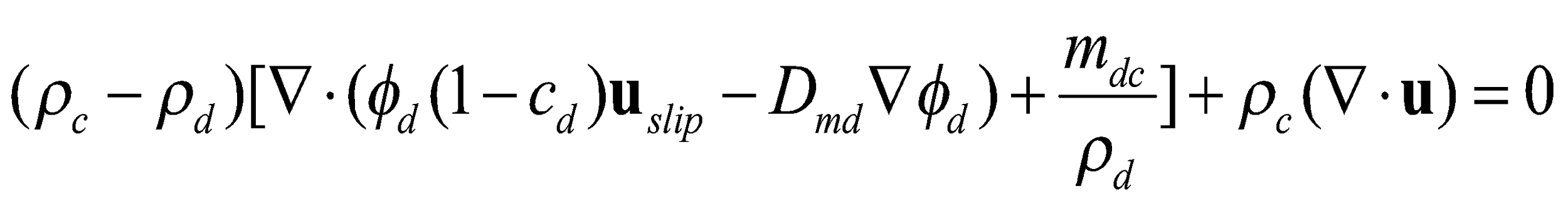_ | 3-4-6-7-10-13 | 21 |  |
| $\rho C_{p}\frac{\partial T}{\partial t}+ \rho C_{p}u\cdot\nabla T+\nabla\cdot q=Q+Q_{r}$ | all | 22 | HEAT TRANSFER WITH RADIATION |
| $q=-k_{c}\nabla T$ | all | 23 |  |
| $Q_{r}=\kappa(G-4\pi I_{b})$ | all | 24 |  |
| $\nabla\cdot\left( D_{P1}\nabla G \right)+\kappa\left( G-4\pi I_{b} \right)=0$ | all | 25 |  |
| $G=\int_{4\pi} I\left( \Omega\right)d\Omega$ | all | 26 |  |
| $D_{P1}=\frac{1}{3\kappa+\sigma_{S}(3-a_{1})}$ | all | 27 |  |
| $\Omega\cdot\nabla I\left( \Omega\right)=\kappa I_{b}\left( T \right)-\beta I\left( \Omega\right)+\frac{\sigma_{S}}{4\pi}\int_{4\pi} I\left( \Omega^{'} \right)\Phi\left( \Omega^{'},\Omega\right)d\Omega'$ | all | 28 |  |
| $\beta=\kappa$ +$\sigma_{S}$ | all | 29 |  |
| $I_{b}\left( T \right)=\frac{n_{r}^{2}\sigma T^{4}}{\pi}$ | all | 30 |  |
| $\frac{I}{I_{0}}=exp(-\beta z)$ | all | 31 |  |
| $\frac{I}{I_{0}}=exp\left( -\frac{3{}_{d}z}{d_{b}}-K_{a}z \right)$ | 6-7 | 32 |  |
| $\mu_{gr}=\frac{\mu_{max}\cdot I}{I+k_{s}+\frac{I^{2}}{k_{i}}}$ | 6-7 | 33 | GROWTH KINETICS |

**Supplementary Table 2 Experimental and simulated light intensities at the outlet of the PBR in abiotic and biotic configurations at increasing incident light intensity.**

|  | ***Case 1*** | | | | ***Case 2*** | | | | ***Case 3*** | | | |
| --- | --- | --- | --- | --- | --- | --- | --- | --- | --- | --- | --- | --- |
|  | **ABIOTIC** | | **BIOTIC** | | **ABIOTIC** | | **BIOTIC** | | **ABIOTIC** | | **BIOTIC** | |
| ***I_s_in*** | ***I_out*** | ***I_out*** | ***I_out*** | ***I_out*** | ***I_out*** | ***I_out*** | ***I_out*** | ***I_out*** | ***I_out*** | ***I_out*** | ***I_out*** | ***I_out*** |
| 50 | 13,7 | 15,2 | 5,0 | 7,5 | 20,5 | 18,3 | 7,0 | 8,5 | 18,1 | 18,6 | 7,6 | 9,67 |
| 100 | 26,5 | 30,5 | 9,7 | 15,5 | 41,4 | 36,6 | 12,8 | 17,1 | 35,7 | 37,2 | 13,7 | 19,3 |
| 200 | 56,6 | 61 | 18,6 | 31 | 80,4 | 73,1 | 26,2 | 34,3 | 71,6 | 74,44 | 28,0 | 38,6 |
| 300 | 87,2 | 91,5 | 28,3 | 46,6 | 122,7 | 109,7 | 46,6 | 50,9 | 113,4 | 111,9 | 43,2 | 58,2 |
| 500 | 148,6 | 152,5 | 61,7 | 77,8 | 207,6 | 182,6 | 95,6 | 94,9 | 188,8 | 186,4 | 90,2 | 96,9 |
| 950 | 287,5 | 289 | 142,7 | 147,7 | 400,8 | 370 | 204,9 | 190,3 | 351,4 | 355,1 | 183,4 | 184,11 |
| 1200 | 362,5 | 366,1 | 178,6 | 186,6 | 504,1 | 494,3 | 250,2 | 256,9 | 435,1 | 450 | 230,7 | 233,1 |
| ***Experimental*** | | ***Model*** | ***Exp.*** | ***Model*** | ***Exp.*** | ***Model*** | ***Exp.*** | ***Model*** | ***Exp.*** | ***Model*** | ***Exp.*** | ***Model*** |

**Supplementary Table 3 Simulated values for the average light perceived and absorbed by bacteria at all the simulated *I_s,n_***

|  | ***_Case 1_*** | | ***_Case 2_*** | | ***_Case 3_*** | |
| --- | --- | --- | --- | --- | --- | --- |
| ***_Is,in_*** | ***_Ip_*** | ***_Iab_*** | ***_Ip_*** | ***_Iab_*** | ***_Ip_*** | ***_Iab_*** |
| _50_ | _17,8_ | _13,6_ | _23,4_ | _16,1_ | _23,9_ | _16,3_ |
| _100_ | _35,8_ | _27,2_ | _46,8_ | _32,1_ | _47,7_ | _32,6_ |
| _200_ | _71,0_ | _54,4_ | _93,7_ | _64,2_ | _95,4_ | _65,4_ |
| _300_ | _107,6_ | _81,6_ | _140,5_ | _96,4_ | _143,5_ | _98,2_ |
| _500_ | _179,4_ | _136,1_ | _234,2_ | _160,3_ | _239,0_ | _163,6_ |
| _950_ | _340,8_ | _258,4_ | _444,8_ | _304,5_ | _454,1_ | _312,15_ |
| _1200_ | _430,5_ | _326,4_ | _633,7_ | _433,8_ | _575,0_ | _395,8_ |
| ***_Experimental_*** | ***_Model_*** | | | | | |

**Supplementary Table 4 Comparisons of efficiency of photosynthesis between the three calibration cases at increasing incident light intensity.**

|  | **I_in_ (umol photons m^-2^ s^-1^)** | **Vol (L)** | **u (h^-1^)** | **mol photons gDW^-1^** | **Efficiency** |
| --- | --- | --- | --- | --- | --- |
| ***Case 1*** | 50 | 0,377 | 0,015 | 3,32 | 0,30 |
|  | 200 | 0,377 | 0,030 | 3,24 | 0,31 |
|  | 300 | 0,377 | 0,054 | 3,65 | 0,27 |
|  | 500 | 0,377 | 0,073 | 4,04 | 0,25 |
|  | 800 | 0,377 | 0,095 | 4,94 | 0,20 |
|  | 950 | 0,377 | 0,099 | 8,18 | 0,12 |
| ***Case 2*** | 50 | 0,377 | 0,022 | 2,12 | 0,47 |
|  | 200 | 0,377 | 0,041 | 2,31 | 0,43 |
|  | 300 | 0,377 | 0,070 | 2,72 | 0,37 |
|  | 500 | 0,377 | 0,089 | 3,10 | 0,32 |
|  | 800 | 0,377 | 0,094 | 4,59 | 0,22 |
|  | 950 | 0,377 | 0,093 | 8,09 | 0,12 |
| ***Case 3*** | 50 | 0,377 | 0,021 | 2,23 | 0,45 |
|  | 200 | 0,377 | 0,041 | 2,28 | 0,44 |
|  | 300 | 0,377 | 0,070 | 2,70 | 0,37 |
|  | 500 | 0,377 | 0,088 | 3,18 | 0,31 |
|  | 800 | 0,377 | 0,097 | 4,53 | 0,22 |
|  | 950 | 0,377 | 0,093 | 8,33 | 0,12 |

**Supplementary Table 5 Model parameters for heat transfer with radiation.**

|  | **Symbol** | **Value** | | **Reference** |
| --- | --- | --- | --- | --- |
| **AIR** | *K_air_* | 0.3 | m^-1^ | (Mogo et al., 2005) |
|  | *S_air_* | 0.00075 | m^-1^ | (Table and Gases, 1974; Fenn et al., 1985; Bucholtz, 1995) |
| **GLASS** | *K_g_* | 3 | m^-1^ | (Erukhimovich and de la Cruz, 2004; refractive index calculator, 2020) |
|  | *S_g_* | 28 | m^-1^ | (JACOBSEN et al., 1971; MIRKO ADEN, ANDREAS ROESNER, 2010) |
|  | *ε_g_* | 0.92 | - | (Mikron Instrument Company, 2014; thermoworks, 2020) |
| **WATER** | *K_w_* | 0.5 | m^-1^ | (Bucholtz, 1995; Pope and Fry, 1997; Downing, 2008) |
|  | *S_w_* | 0.008 | m^-1^ | (Downing, 2008; Röttgers et al., 2010) |
| **STAINLESS STEEL** | *ε_s_* | 0.96 | - | (Mikron Instrument Company, 2014; thermoworks, 2020) |
